# Application of nanotechnology in the treatment of hepatocellular carcinoma

**DOI:** 10.3389/fphar.2024.1438819

**Published:** 2024-11-29

**Authors:** Liu Cai, Yanyuan Du, Hongtai Xiong, Honggang Zheng

**Affiliations:** Department of Oncology, Guang’anmen Hospital, China Academy of Chinese Medical Sciences, Beijing, China

**Keywords:** hepatocellular carcinoma, nanotechnology, tumor, anti-tumour mechanism, immune function

## Abstract

Hepatocellular carcinoma is the predominant histologic variant of hepatic malignancy and has become a major challenge to global health. The increasing incidence and mortality of hepatocellular carcinoma has created an urgent need for effective prevention, diagnosis, and treatment strategies. This is despite the impressive results of multiple treatments in the clinic. However, the unique tumor immunosuppressive microenvironment of hepatocellular carcinoma increases the difficulty of treatment and immune tolerance. In recent years, the application of nanoparticles in the treatment of hepatocellular carcinoma has brought new hope for tumor patients. Nano agents target tumor-associated fibroblasts, regulatory T cells, myeloid suppressor cells, tumor-associated macrophages, tumor-associated neutrophils, and immature dendritic cells, reversed the immunosuppressive microenvironment of hepatocellular carcinoma. In addition, he purpose of this review is to summarize the advantages of nanotechnology in guiding surgical excision, local ablation, TACE, standard chemotherapy, and immunotherapy, application of nano-vaccines has also continuously enriched the treatment of liver cancer. This study aims to investigate the potential applications of nanotechnology in the management of hepatocellular carcinoma, with the ultimate goal of enhancing therapeutic outcomes and improving the prognosis for patients affected by this malignancy.

## 1 Introduction

Hepatic cancer is the fifth most frequently occurring type of malignancy across the globe, is now a major contributor to the global cancer burden (Anwanwan et al., 2020). Hepatocellular carcinoma (HCC) is the most common histological type of liver cancer, accounting for more than 80% of all cases. HCC is 2–3 times more common in men than in women, with a more pronounced difference in European countries (Nevola et al., 2023). Risk factors for HCC include non-alcoholic fatty liver disease (NAFLD)/non-alcoholic steatohepatitis (NASH), hepatitis B virus (HBV), and hepatitis C virus (HCV) (Brown et al., 2023). In addition to cirrhosis, alcoholic liver disease (ALD) can also result in aberrant liver metabolism and HCC (Persson et al., 2013). In addition, some researchers have found that smoking is another important factor causing HCC. Studies have shown that smokers have a 47%–86% increased risk of HCC, and HCC risk levels almost return to baseline after 30 years of quitting. Heavy drinkers have a 68%–87% increased risk of HCC (Petrick et al., 2018). This may be due to the mutagenic effect of acetaldehyde and the increased risk of HCC by reactive oxygen species produced by excess iron deposition in the liver. Alcohol has also been reported to accelerate hepatitis C virus-induced liver tumorigenesis through the Toll-like receptor (TLR4) signaling pathway (Taniai, 2020). Aflatoxin B1 metabolites in the liver have been shown to covalently bind to guanine bases on hepatocyte DNA molecules at N7, interfering with normal DNA transcription and forming AF-DNA adducts. In South America and Southeast Asia, where aflatoxin exposure is higher, the risk of HCC is 70 times higher (Yang et al., 2017).

Hepatocellular carcinoma frequently presents at an early stage with vague symptoms, combined with the absence of reliable early diagnostic markers, leading to the majority of cases being identified at intermediate to advanced stages (Kobayashi et al., 2017). Barcelona Clinic Liver Cancer (BCLC) is the most widely accepted analyzing system in the world and has been confirmed in a large number of clinical studies. The BCLC clinical staging system for hepatocellular carcinoma classifies the disease into five distinct categories: very early stage, early stage, intermediate stage, advanced stage, and end-stage (Llovet et al., 2004). For patients in the earliest stages of HCC,and surgical interventions such as hepatectomy and liver transplantation are crucial for achieving long-term survival. However, only 20%–30% of patients qualify for surgical resection, and the 5-year recurrence rate post-resection ranges from 40% to 70%. Therefore, patients who undergo surgical resection require close follow-up. For intermediate and advanced patients who cannot undergo surgical treatment or who have recurrent disease after surgery. According to the characteristics of recurrent diseaseLocal ablation, transarterial chemoembolization (TACE), radiation therapy or systemic therapy can be implemented to help patients prolong their survival (Zhou et al., 2020). Sorafenib, a kinase inhibitor, is frequently used by patients with advanced HCC to suppress tumor growth and metastasis. Nonetheless, only approximately one-third of patients experience beneficial effects from this treatment. Furthermore, prolonged use of sorafenib is associated with issues such as cytotoxicity and the development of drug resistance, There is no effective treatment for end-stage patients, in which case only palliative care is used to alleviate the patient’s suffering (Keating, 2017). Therefore, further research is needed to find better ways to treat HCC.

Nanoparticles (NPs) are generally characterized as particles that measure less than 100 nm. They can be classified according to their shape, size, and chemical characteristics, with common varieties including carbon-based NPs, metallic NPs, magnetic NPs, semiconductor NPs, polymeric NPs, and lipid-based NPs (Khan et al., 2019). Currently, nanoparticles are extensively utilized in diagnosing and treating genetic disorders, autoimmune diseases, malignant tumors, and various other medical conditions (Sukhanova et al., 2018). Alzheimer’s disease and Parkinson’s disease are significant neurodegenerative disorders that pose major challenges for the medical field to tackle. The blood-brain barrier (BBB) is one of the most important barriers to the development of new therapeutic agents and biologics for the central nervous system (CNS). Several studies have found that nanotechnology can assist drugs in reaching target tissues through the BBB, bringing new hope to Alzheimer’s disease and Parkinson’s patients (Li et al., 2021; Song N. et al., 2023; Unnisa et al., 2023). Nanomaterials are also widely used in dental diseases because of their good mechanical properties, wear resistance, antimicrobial activity and many other advantages (Malik and Waheed, 2023). Furthermore, advancements in nanotechnology have demonstrated considerable promise in addressing viral infectious diseases, particularly respiratory viral infections. Engineered nanocarriers have played a crucial role in the targeted delivery of drugs and vaccines, significantly improving therapeutic efficacy against viral pathogens (Seyfoori et al., 2021). With the help of nanotechnology, ophthalmic diseases are now being revolutionized in terms of drug delivery and post-operative scar repair. Compared with traditional drug delivery methods, nanocarriers extend the residence time of drugs, reduce drug degradation, decrease the frequency of drug administration, and improve patient compliance. And the nanoemulsion drug delivery system also shows obvious advantages in gene therapy and exosomes (Liu et al., 2023). In recent years, the rapid progress in nanotechnology and materials science has led to the integration of nanoparticles into many common diagnostic and therapeutic approaches for HCC (Graur et al., 2022). NPs plays an important role in intraoperative imaging, local ablation, TACE, immunotherapy and other aspects of HCC due to its small size, large area-to-volume ratio and specific physical properties (Wu et al., 2021). This is mainly due to the development of nanomaterials in the treatment of HCC in drug delivery, specific targeting, enhanced drug efficacy, multi-drug combination, combined imaging methods to assist surgery, and visualization of drug delivery play a strong advantage (Bakrania et al., 2021). Here, nanoreagents have been actively used in the clinic (Table 1). This suggests that advances in nanotechnology offer more possibilities for multidisciplinary diagnosis and treatment of HCC.

**TABLE 1:** Clinical application of nanomaterials in HCC.

Nanomaterials	Intervention	Research object	Trial group	Control group	Intervention time	Main outcome indicators	References
PBCA-NPs	Intravenous administration of mitoxantrone	unresected HCC	DHAD-PBCA-NPs	DHAD		stable disease: 61.4% v.s.45.1% (*P* < 0 .05)	Zhou et al. (2009)
Tpgs	targeted nanoparticle delivery system for sorafenib (SFB)	HCC	TACE and Ab-SFB-NP system	TACE and non-nano drug delivery system	3months	The AST after 3 months treatment: (52.21 ± 26.01) U/Lv.s. (36.00 ± 14.00) U/L; the DCR after 3 months treatment:88.9% v.s. 58.4% (P< 0 .05)	Su (2021)
SHIFT&Indocyanine green nanoparticles (SHIFT&nanoICG)	transhepatic arterial embolization combined with fluorescent laparoscopic hepatectomy	liver cancer				The method effectively identifies and marks lesions with remarkable stability, embolic properties, optical imaging capabilities, and an improved tumor-to-normal tissue ratio. This is particularly beneficial for detecting microsatellite lesions (0.4 × 0.3 cm) that preoperative imaging could not identify, enabling complete resection of hepatocellular carcinoma under fluorescence laparoscopy in a shorter time frame (within 2 h) and with reduced intraoperative blood loss (50 mL)	He et al. (2022)
TKM-080301	intravenous infusion	Advanced HCC	TKM-080301 as an intravenous infusion	sorafenib	8 months	median OS:7.5months v.s. 6.5 months	El Dika et al. (2019)
Iron oxide nano-particle m-PEG-silane (IOP) Injection	MR imaging	suspected HCC	IOP-enhanced MRI	the pathology reports	5d	IOP Injection enhanced the lesion-to-liver contrast ratio in T2 *-weighted images by 50.1% ± 4.8%. IOP-enhanced MRI detected HCC with 100% sensitivity by subject and 96% sensitivity by lesion	Chiang et al. (2023)
SHU-555-A	magnetic resonance (MR) image sequences	①cirrhotic liver of variable degree ②all showed suspected HCC with or without intrahepatic metastases	Contrast + SHU-555-A	Contrast	2 weeks	Tumors were better detected after injection of SHU-555-A on all pulse sequences except on out-of-phase T1-weighted (T1W)-GRE sequences	Arbab et al. (2000)
SPIO	magnetic resonance (MR) imaging	histologically proved HCCs associated with liver cirrhosis	superparamagnetic iron oxide (SPIO)-enhanced	Unenhanced magnetic resonance (MR) imaging		When using SPIO-enhanced SE and FLASH sequences, lesions were identified more accurately compared to unenhanced images (*P* < 0.05)	Yamamoto et al. (1995)
ferucarbotran	ferucarbotran-enhanced magnetic resonance (MR) imaging	representative hepatic lesions	well differentiated HCC	moderately differentiated HCC, poorly differentiated HCC		T2 PSIL:39.5% ± 8.23% v.s.26.4% ± 13.78% v.s.4.4% ± 9.42% (*p* < 0.001)	Chen et al. (2008)
ferumoxides	MR imaging	HCC	ferumoxides-enhanced	mangafodipir trisodium—enhanced		the mean sensitivities of MR imaging: 92% v.s. 81% (*p* < 0.05)	Kim et al. (2002)
AMI-25	MR imaging	hepatic tumors	AMI-25-enhanced			141/159 cases (88.7%) were evaluated “useful” or “very useful” for clinical usefulness. For efficacy including the signal to noise ratio (S/N) of liver and the tumor-liver contrast to noise ratio (C/N), 89.6% of hepatocellular carcinomas and 95.0% of metastatic liver cancers were evaluated “effective” or “very effective”, respectively	Yoshikawa et al. (1994)
USPIO	MR imaging	Focal liver disease	Ultrasmall superparamagnetic Iron Oxide (USPIO)	small superparamagnetic Iron Oxide (SPIO)		Both SPIO andUSPI0 provided >500% improvement in LLC on T2WIOn TIWI, LLC was increased in metastases (120%) andHCC(325%) with SPIO, Post-USPIO, LLC was increasedon TIWI only in metastases (>500%) (*p* < 0.05)	Mergo et al. (1998)
Code-7227	MR imaging	HCC	after intravenous administration of Code-7227	before intravenous administration of Code-7227		The level of enhancement observed in T1-weighted images and the reduction in signal intensity on T2-weighted images were notably less in malignant liver masses compared to hemangiomas. (*p* < 0.001)	Harisinghani et al. (1997)

This paper initially discusses the distinct immunosuppressive microenvironment of HCC, where various immune cells interact and contribute to the disease’s progression. Then, it introduces the shortcomings of various current treatment methods in HCC, and the advantages of NPs in intraoperative imaging, local ablation, TACE, standard chemotherapy, and immunotherapy in HCC. Finally, it summarizes the shortcomings of NPs in the current clinical application. Our purpose is to gain insight into how to design more effective nanoplatforms to ultimately improve treatment and prognosis for HCC patients.

## 2 Hepatocellular carcinoma immunosuppressive microenvironment

**FIGURE 1:**
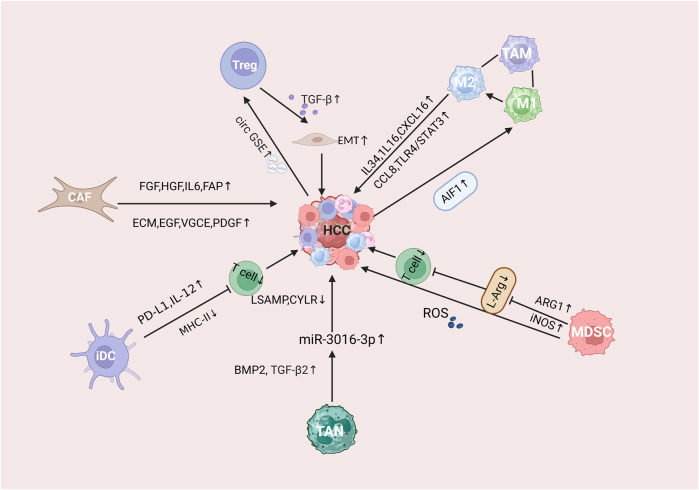
Mechanistic description of the immunosuppressive microenvironment of HCC tumors. Where CAFs refers to tumor-associated fibroblasts, Treg refers to regulatory T cells, MDSCs refers to myeloid-derived suppressor cells, TAM refers to tumor-associated macrophages, TAN refers to tumor-associated neutrophils, and iDC refers to immature dendritic cells.

Liver is the largest internal organ in the human body. Due to its anatomical location and tissue structure, the microenvironment of HCC shows stronger immunosuppression compared with other tumors, forming a unique immunosuppressive environment (Yin et al., 2024). The liver is not only an important part of the defense against blood-borne infections, but it also constantly clears the microorganisms, compounds and pathogens in the gut, so that it is protected from intestinal antigens secondary immune damage.It is called “immunologically privileged organ” (Jenne and Kubes, 2013). HCC is frequently considered a malignancy linked to inflammatory processes, where the tumor’s immunosuppressive microenvironment is crucial in its pathogenesis, progression, and response to anti-tumor immunotherapy (Miao and Nan, 2022). In the HCC immune microenvironment, a variety of immunosuppressive cells, including tumor-associated macrophages (TAM), bone marrow-derived suppressor cells (MDSC), tumor-associated neutrophils (TAN), cancer-associated fibroblasts (CAF), regulatory T cells (Tregs), and dendritic cells (DCs), The progression of HCC is facilitated through a series of complex pathways that play a role together (Lu et al., 2019) (Figure 1).

TAMs are key players within the immunosuppressive cellular and cytokine network, significantly contributing to tumor immune evasion mechanisms (Goswami et al., 2021). TAMs are abundant in HCC, and in HCC, TAMs mostly favor the M2 phenotype. With the deepening of the understanding of TEM, TAM with immune heterogeneity has become a hot topic for researchers to discuss (Elliott et al., 2017; Huang et al., 2021). Research has shown that M2-associated macrophages can enhance the proliferation of HCC cell lines via the TLR4/STAT3 pathway (Yao et al., 2018). The influence of TAMs on HCC proliferation and metastasis involves the chemokine (C-X-C motif) ligand 8 (CXCL8) (Yin et al., 2017). Studies have indicated that allograft inflammatory factor 1 (AIF1) is exclusively expressed in TAMs within the HCC microenvironment. AIF1 overexpression not only drives macrophage polarization towards the M2 phenotype but also facilitates HCC cell migration through the secretion of CXCL16 (Cai et al., 2017). In addition, IL-6 derived from TAMs under hypoxic conditions is also believed to promote HCC metastasis and invasion (Deng et al., 2021). MicroRNAs (miRNAs), a category of small non-coding RNAs, serve as essential regulators of tumor metastasis in hepatocellular carcinoma (HCC). Research has indicated that a deficiency in miR-28-5p is inversely associated with the expression of interleukin-34 (IL-34) and the infiltration of TAMs, with elevated IL-34 levels further promoting HCC progression (Zhou et al., 2016).

DC dysfunction often synergizes with other mechanisms to promote HCC development and immune escape of tumor cells (Wang S. et al., 2022). Most studies have demonstrated that immature dendritic cells result in an attenuated anti-tumor immune response due to their inability to present relevant antigens to T cells (Chen et al., 2000). The capacity of immature dendritic cells to produce cytokines, such as interleukin-12 (IL-12), is diminished, leading to reduced activation of T-cell-mediated immune responses. This, in turn, facilitates the development of a tumor-supportive immunosuppressive microenvironment. Extensive research has demonstrated that within the HCC tumor microenvironment, hypoxia and factors like PGE2 and TGF-β, secreted by tumor cells, induce Kupffer cells to release interleukin-10 (IL-10), further contributing to immune suppression (Shiri et al., 2024). It has been demonstrated that immature dendritic cells secrete more IL-10, and the released IL-10 cytokines can also indirectly recruit MDSCs, which in turn can promote the transformation of dendritic cells into tolerogenic dendritic cells, facilitating the emergence of organismal tolerance to HCC (Oura et al., 2021). Moreover, DCs have the ability to promote Treg differentiation. In a subset of HCC patients, dendritic cells exhibit increased expression of PD-L1 on their surface. This upregulation allows PD-L1 to interact with its receptor on T cells, thereby inhibiting the activation of antigen-specific T cells and further contributing to immune evasion (Chen C. et al., 2023). The above multiple mechanisms interact with each other in the tumor immunosuppressive microenvironment, and together they promote the growth and proliferation of HCC.

MDSCs a class of inhibitory cells originating from bone marrow, serve as precursors to dendritic cells (DCs), macrophages, and granulocytes. They possess a strong capacity to suppress immune cell responses (Umansky et al., 2016). Research shows that that MDSCs exert powerful immunosuppressive effects on HCC through various mechanisms (Tomiyama et al., 2022). The activities of ARG1 and iNOS enzymes are key factors in MDSC-induced immunosuppression. In both murine models and HCC patients, the expansion of MDSCs significantly enhances the secretion of arginase 1 (ARG1) and inducible nitric oxide synthase (iNOS). This leads to a depletion of L-arginine, an amino acid that is conditionally essential for T cell function, consequently impairing T cell differentiation and proliferation (Wang Y. et al., 2021). The release of reactive oxygen species (ROS) is also one of the main ways that MDSCs inhibit T cell activity. We found that the overproduction of ROS caused MDSCs to induce nitration of TCR/CD8, which directly disrupted the binding of specific peptide-MHC (pMHC) dimers to CD8T cells (Nagaraj et al., 2007).

Multiple studies have shown that elevated neutrophil infiltration is strongly correlated with poor tumor prognosis (Que et al., 2022). However, their function within the tumor microenvironment remains a subject of debate (Granot and Jablonska, 2015; Sagiv et al., 2015; Sionov et al., 2015). As insights into the tumor microenvironment have expanded, it has been established that neutrophils directly facilitate tumor growth by secreting an array of cytokines and chemokines, which perpetually recruit them into the tumor microenvironment (Shaul and Fridlender, 2018). In addition, TNA has also been identified as playing a key role in tumor angiogenesis. And promote the migration and invasion of tumor cells by secreting enzymes that modify and degrade the extracellular matrix (Granot and Fridlender, 2015). A recent study revealed that TANs can secrete bone morphogenetic protein 2 (BMP2) and TGF-β2, triggering the expression of miR-301b-3p in HCC cells within the tumor microenvironment. This finding clarifies the direct regulatory role of TANs in miR-301b-3p regulation. Consequently, the stem cell-like properties of HCC cells are enhanced by inhibiting the gene expression of limbic system-associated membrane protein (LSAMP) and CYLD lysine 63 deubiquitinase (CYLD) (Zhou et al., 2019).

CAF, as a group of heterogeneous dynamic fibroblasts, differs from normal fibroblasts and can infiltrate tumor cells (Simon and Salhia, 2022). In addition, in terms of structure and function, CAFs can provide a fertile environment for tumor growth by secreting extracellular matrix ECM protein, epidermal growth factor (EGF)/fibroblast growth factor, pro-angiogenic factor, platelet-derived growth factor (PDGF), chemokines and other factors (Zhang J. et al., 2020). As the main components of HCC tumor matrix,. As the main components of HCC tumor matrix, CAFs enhance the dry of CD24^+^ cells and promote HCC progression by activating HGF and IL6 secreted by STAT3 Tyr705 phosphorylation (Li et al., 2019). HCC is a highly vascularized tumor. Research has demonstrated that CAFs regulate the EZH2/VASH1 pathway by secreting VEGF, which in turn promotes the proliferation and angiogenesis of human umbilical vein endothelial cells. This finding further highlights the role of CAFs in HCC proliferation and angiogenesis (Huang et al., 2019). Additionally, increasing evidence shows that CAFs significantly shape the immunosuppressive microenvironment. Fibrocyte activating protein (FAP) activates the uPAR-FAK-c-Src-JAK2 signaling cascade, which subsequently triggers STAT3, leading to the formation of inflammatory CAFs. CCL2 secreted by FAP + CAFs enhances the recruitment and immunosuppressive activity of MDSCs through CCR2, thereby promoting tumor proliferation and invasion (Yang et al., 2016).

Tregs as a group of T cells with unique functions, are generally considered to promote tumor growth (Kobayashi et al., 2007). Based on previous studies, the increase of CD4^+^ and CD8^+^ TREgs is closely related to immune impairment in HCC patients (Fu et al., 2007). It has been discovered that Tregs can induce epithelial-mesenchymal transition (EMT) through transforming growth factor-β1 (TGF-β1), thereby promoting the invasion and migration of Hepa1-6 cells (Shi et al., 2019). In addition, quantitative RT-PCR in a recent study found that the HCC-derived exosome circGSE1 enhances HCC proliferation, migration, and invasion by inducing Tregs amplification (Tang et al., 2022). Furthermore, clinical studies have demonstrated that poorly differentiated HCC patients exhibit a significantly higher ratio of Tregs in peripheral blood compared to those with highly differentiated HCC. This elevated Treg ratio is closely associated with tumor recurrence and lower survival rates in HCC patients (Zhou et al., 2021).

## 3 Nano-agents reverse the immunosuppressive microenvironment of HCC

Despite significant advancements in immunotherapy for malignant tumor treatment in recent years, its limited effectiveness and considerable toxicity continue to present major challenges in managing HCC today. The development of an immunosuppressive microenvironment is a crucial factor contributing to the poor therapeutic outcomes in HCC patients undergoing ICIs. With an expanding understanding of this immunosuppressive milieu in HCC, nanomaterials have been identified as a vital approach to reverse it through the targeted delivery of immunopharmaceuticals or the implementation of multimodal synergistic therapies.

The interplay between TAM and immune cells is pivotal in shaping the immunosuppressive microenvironment of HCC. Currently, researchers mostly reverse the tumor immunosuppressive microenvironment in three ways: directly killing TAMs, inhibiting or blocking monocyte recruitment to tumor tissues, and inducing repolarization of TAMs toward an anti-tumor M1 phenotype (Zhu et al., 2020). In order to address the off-target effect of free drugs in immunotherapy, Wang et al.developed a novel co-delivery system of CMCS/SF-CLN and CMCS/M-IMD-CLN called twin-like core-shell nanoparticles (TCN). This system has the ability to target TAM while being able to localize tumors for chemo-immunotherapy (Wang et al., 2019). Hang et al. developed a galactosylated cationic dextran (gal-C-dextran) and CpG oligodeoxynucleotides (ODN) combined to form a stable nanocomplex (GDO, gal-C-dextran + ODN)) for immunotherapy of hepatocellular carcinoma. The system was tested separately in an *in vivo* and *in vitro* model of hepatocellular carcinoma. The results showed that gal-C-glucan + ODN could be precisely targeted into TAM without affecting systemic immunity and effectively redirected the polarization of these macrophages, exhibiting significant tumor-killing activity (Huang et al., 2012). Han et al. (2023) used M2 macrophage-binding peptide (M2pep) modified poly (propylene cross ester-ethylene cross ester copolymer) nanoparticles loaded with d-lactate (DL; a gut microbiome metabolite) to form a new nanopreparation (DL@NP-M-M2pep)It was injected multiple times in a Hepa1-6-luc-derived *in situ* HCC mouse model. In comparison to free DL, DL@NP-M-M2pep significantly suppressed tumor progression and enhanced the survival rates of mice over a 21-day period. Furthermore, utilizing specific markers for M2 and M1 TAMs, it was confirmed that DL@NP-M-M2pep markedly reduced the number of M2 TAMs while increasing the population of F4/80 CD86 M^1^ macrophages within the tumors, compared to PBS and free DL treatments. All of the above studies have demonstrated that TAM-specific immunomodulation by nano-agents may be valuable for current HCC immunotherapy.

DC maturation is essential for the activation of anti-tumor effector T cells, thereby reversing the immunosuppressive microenvironment associated with tumors (Wculek et al., 2020; Hato et al., 2024). Xiao et al. aimed to effectively activate tumor-infiltrating dendritic cells (TIDC) to enhance the anti-tumor immune response following immune checkpoint blockade (CIB) after HCC ablation. The team incorporated an inhibitor targeting the fat mass and obesity-associated gene (FTO) into a nanocarrier. Subsequently, M/m-MP@F captured antigens from lysates of tumor cells, resulting in final nanodrugs designated as M/Ag-m-MP@F. Results from RT-qPCR and Western blot analyses on bone marrow-derived dendritic cells treated with M/Ag-m-MMP@F indicated a notable increase in the expression levels of co-stimulatory molecules CD80, CD86, and MHC-II. Additionally, flow cytometry revealed that the maturation level of DCs treated with M/Ag-m-MP@F reached 68.6%, significantly surpassing the control group’s 13.5% (Xiao et al., 2023). In addition, Yang et al. developed a nanodelivery system, CpG/DOX-B-PDA (CDBP), utilizing boronophenylalanine-modified polydopamine (PBA-PDA) nanoparticles that were loaded with doxorubicin and the immunosuppressant CpG oligodeoxynucleotides. The findings indicated that this system could effectively infiltrate deep into hepatocellular carcinoma (HCC) tissues and be absorbed by tumor cells, thereby enhancing the efficacy of chemotherapeutic agents in targeting these cells. Additionally, this drug delivery mechanism can work synergistically with photothermal therapy to trigger the release of tumor antigens, creating an “eat-me” signal alongside the innate immune agonist CpG-ODN, which facilitates the formation of an *in situ* vaccine. This process promotes both proliferation and activation of dendritic cells (DCs) as well as downstream CD8 T-cells, ultimately aiding in the prevention of HCC recurrence and metastasis (Yang et al., 2024). Zhang et al. developed a lipid nanoparticle (RNA LNP) that encapsulates tumor RNA and successfully targeted the delivery of this RNA to the tumor site. In both *in vivo* and *in vitro* models of HCC, it was demonstrated that the RNA LNP vaccine effectively inhibited HCC growth by enhancing DC maturation, which subsequently stimulated T lymphocytes to eliminate tumor cells (Zhang Y. et al., 2021).

Clinical study data indicate that increased levels of MDSCs in the peripheral blood and tumor tissues of patients with HCC significantly contribute to their recurrence and unfavorable prognosis (Tan et al., 2019). Tang et al.In order to reduce the risk of recurrence in HCC patients due to in cases ofinsufficient RFA (iRFA) postoperatively. A novel size-tunable nanoliposome delivery of MDSCs inhibitor (IPI549) and αPDL1 antibody (LPIP) was developed, and LPE @PFH@IPI549 (LPI) and LPE@PFH@IPI549@*α*PD-L1 (LPIP) nanoparticles were successfully prepared. The nanoparticles not only successfully inhibited the survival of MDSCs and blocked the compensatory expression of PDL1 on MDSCs by releasing immunomodulators in the transition region of iRAF, but also provided a new strategy for HCC eradication by RAF (Tang et al., 2023).

The immune “cold” state of HCC is a significant cause of the tumor’s poor immune response (Donne and Lujambio, 2023). In order to reverse the “cold” state of tumor immunity to a “hot” state, TAN are remodeled to enhance their immunotherapy. Wang and colleagues incorporated captopril complexes into silicon phthalocyanine dichloride, which was embedded in mesoporous silica (SMN). Following stimulation with specific neoantigens in H22 mice, they harvested mature dendritic cells (mDCs). The membranes of these mDCs were then stripped and applied to the surface of SMNs to create the mD@cSMNs vaccine. The results indicated that treatment with mD@cSMNs significantly suppressed tumor growth in a primary mouse model of HCC. Further analysis showed a notable decrease in tumor-promoting N2 neutrophils within the experimental group. Additionally, flow cytometry revealed that mD@cSMNs facilitated the polarization of tumor-associated neutrophils from the N2 phenotype to the N1 phenotype, effectively reversing the immunosuppressive microenvironment associated with HCC (Wang Y. et al., 2022).

Nanoformulations application can inactivate collagen-producing CAF thereby cutting off the source of matrix proteins, demonstrating that the reduction of ECM deposition can be achieved by directly or indirectly modulating CAF to prevent and control the occurrence of abnormal ECM (Niveria et al., 2022). JianGuo et al. found that CFH peptide (CFHKHKSPALSPVGGG)-decorated liposomal oxymatrine (CFH/OM-L) could directly inactivate CAF by reversing epithelial-mesenchymal transition. Researchers applied lipid complexes containing Epimedium to a xenograft model of hepatocellular carcinoma in nude mice. The results showed that the lipid complex not only directly killed CAFs and effectively reversed the ETM process *in vivo*, but also promoted the polarization of M1 tumor-associated macrophages and re-edited the tumor microenvironment, which provided favorable conditions for the in-depth study of nanoparticles (Guo et al., 2022). YuXiao et al. designed a nanoparticle of polylactic acid-hydroxyacetic acid copolymer modified by mannan to be used as a drug-carrying medium for simvastatin. This nanocarrier system directly attenuates capillarization of liver sinusoidal endothelial cells (LSECs) (Yu et al., 2022). In addition, a recent study showed that nanomedicines can indirectly affect CAF activation and function by targeting LSECs in hepatocellular carcinoma, which can remodel the HCC tumor microenvironment (Pan et al., 2024).

The tumor microenvironment induced by Treg cells is regarded as a crucial mechanism facilitating immune evasion by tumors (Curotto de Lafaille and Lafaille, 2009) Ou et al. (2018) created hybrid nanoparticles conjugated with the tLyp1 peptide, which improved the ability of imatinib to downregulate Treg cells in the tumor microenvironment by inhibiting the phosphorylation of STAT3 and STAT5, leading to an anti-tumor effect. Yu et al. (2020) to enhance the impact of immunogenic cell death (ICD) in HCC, researchers developed poly lactic-co-glycolic acid (PLGA)-polyethylene glycol (PEG)-aminoethyl anisamide (AEAA) nanoparticles for the targeted co-delivery of icaritin and doxorubicin. Experimental findings revealed that treatment with PLGA-PEG-AEAA nanoparticles significantly reduced levels of immunosuppressive cells, such as MDSCs, Tregs, and M2 macrophages, in a Hepa1-6 inoculated mouse model of HCC. This reduction effectively restructured the immunosuppressive microenvironment, resulting in inhibited progression of HCC.

## 4 Application of nanotechnology in hepatocellular carcinoma

**FIGURE 2:**
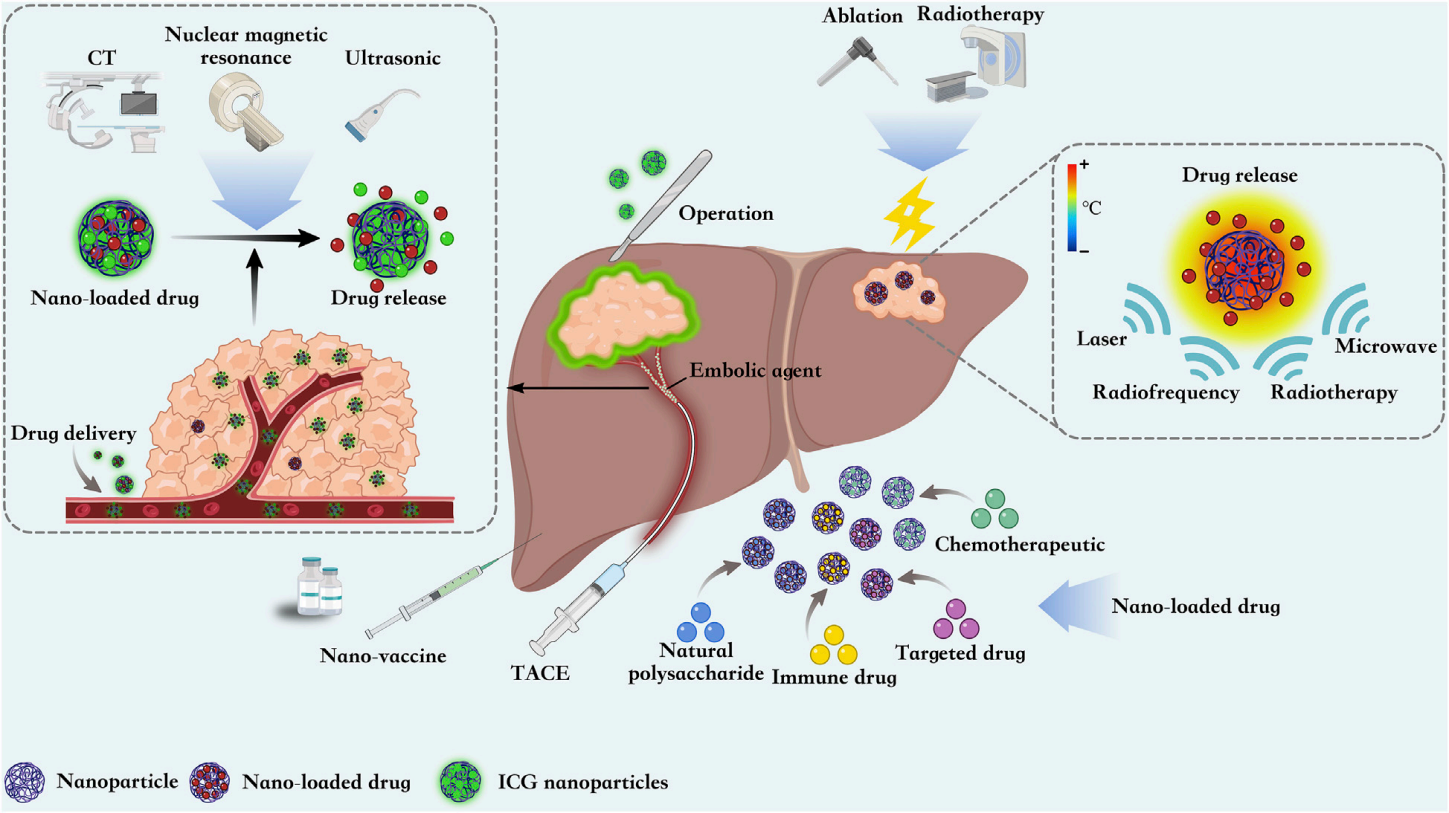
Nanoparticles are able to guide surgical resection of HCC by loading ICG contrast agents, They can also be loaded with chemotherapeutic agents as embolic agents for TACE. Nanoparticles focuses energy from external sources on the tumor site, thereby inducing local tumor destruction and improving ablation efficiency. At the same time, nanoparticles can also be loaded with chemotherapeutic, immunologic, and targeted drugs and natural polysaccharides for precise targeting of HCC. The development of multiple nanovaccines also offers new strategies for the treatment of HCC.

Nanomaterials hold significant potential for the treatment of liver cancer, attributed to their distinctive acousto-opto-electromagnetic physical characteristics and stable chemical properties. The transfer of fluorophores by nanotechnology in HCC surgery can help doctors to clearly locate the tumor, thus reducing the amount of blood loss and achieving accurate excision. In addition, nanoparticles also show great potential in ablation, radiotherapy and TACE. The widespread use of drug delivery and nanovaccines in HCC also holds promise for prolonging the survival of HCC patients (Figure 2).

### 4.1 Application of HCC surgery

For most patients with solid tumors, surgical resection remains the most crucial and effective treatment option (Bortot et al., 2023). However, the distinction between tumor and healthy tissue based on touch, vision, and the surgeon’s experience has certain limitations. Fortunately, intraoperative fluorescence imaging technology has brought hope for accurate surgical resection of HCC patients due to its advantages of high contrast, low cost and real-time feedback (Verbeek et al., 2012). Fluorophores with near-infrared fluorescence (NIR) have a higher penetration range than fluorophores emitting short wavelength electromagnetic radiation (Frangioni, 2003). Indocyanine green (ICG) is the only NIR fluorophores approved for intraoperative guidance. However, it has been found in HCC surgical practice that ICG greatly limits its clinical application due to its rapid clearance in blood, poor stability and lack of tumor specificity (Zhang et al., 2017; Tang Z. et al., 2020). The continuous development of nanotechnology and biomaterials has expanded their bioavailability and provided the possibility to overcome the problems of ICG in HCC surgery. For example, lipid-like nanovesicle-loaded ICG nanoparticles showed longer blood circulation and tumor specificity (Zhang et al., 2018). Nanotechnology has shown great potential in image-guided surgery (IGS) therapy for HCC (Wojtynek and Mohs, 2020).

Jin et al. (2024) developed a nanoparticle drug delivery system based on SP94-modified hollow Fe3O4(SP94-Fe3O4@ICG) compared SP94-Fe3O4@ICG with MPI contrast agent (Vivotrax) at different concentrations. The results show that the two-dimensional projected MPI image of SP94-Fe3O4@ICG is much higher than that of Vivotrax at the same concentration. These results indicate that the application of SP94-Fe3O4@ICG provides a possibility for accurate detection and surgical navigation in HCC surgery.

To ensure the uniform dispersion, long-term stability, and high fluorescence intensity of ICG, which allows for precise identification of tumor margins. Zhang et al. (2022) developed carrier-free ICG nanoparticles utilizing super-stable homogeneous lipiodol formulation technology, referred to as SHIFT nanoICG, for precise surgical interventions following TAE. In the VX2 *in situ* hepatocellular carcinoma model, researchers employed SHIFT nanoICG for accurate surgical navigation of HCC 7 days post-TAE treatment. As expected, SHIFT nanoICG exhibited specific fluorescence within tumor tissues, clearly delineating tumor regions and boundaries while effectively differentiating between tumor and normal tissue, thereby aiding in guided liver resection. This study demonstrated that SHIFT nanoICG possesses excellent stability, homogeneity, drug release characteristics, and embolic effects. Notably, its ultra-high stability was maintained for over 60 days. These findings underscore the remarkable biocompatibility, safety profile, and clinical potential of SHIFT nanoICG.

Powerful FL intensity and excellent resistance to photobleaching are key to successful surgery for hepatocellular carcinoma. Zhu et al. (2022) prepared ICG-thiodinol nanoemulsions by self-emulsification method, and their characterization was appropriately modified to make their optical properties more stable. After the *in situ* liver tumor model was established, mice were randomly divided into nanoemulsion and ICG groups. The nanoemulsion and ICG (2 mg/kg) were injected intravenously and surgical resection was performed at 24 h. *In vivo* studies revealed that nanoemulsion-modified ICG exhibited stronger FL strength compared to free ICG. On day 5, the FL signal of free ICG decreased to 0, whereas the FL signal intensity of nanoemulsion-prepared ICG was still greater than 50% on day 6. These demonstrated that the application of nanoemulsion could accurately highlight the tumor lesions during the surgery of HCC, thus guiding the effective intraoperative resection of hepatocellular carcinoma.

He et al. (2022) improved the photothermal stability of ICG molecules through the supersurface pure nanomedical preparation technology, which effectively avoided the rapid decomposition of ICG *in vivo*. ICG was uniformly mixed into thiiodol fluorescent-guided surgical navigation using the SHIFT. It was found that nanoICG exhibited excellent tumor-specific deposition after integration with lipoiodol by SHIFT, and nanoICG exhibited 3 times more photobleaching resistance than free ICG molecules. On this basis, HePan et al. recruited a 52-year-old HCC patient for a clinical trial. The results show that SHIFT&nanoICG can accurately identify tumor lesions, and complete surgical resection of HCC can be achieved under fluorescence laparoscopy with shorter time and less blood loss.

Intraoperative bleeding in HCC is a major problem in hepatectomy, which not only affects the surgical outcome but also significantly increases the postoperative recurrence rate. Therefore, the amount of blood loss and the possibility of blood transfusion should be minimized during the operation. Zhu et al. (2024) used a polyethylene glycol (PEG) block copolymer as a drug protective carrier for altezumab and bevacizumab, and then synthesized a nanocomposite hemostatic hydrogel using nanotechnology. In addition, the stability of monoclonal antibody drug calcium magnesium carbonate nanoparticles prepared by chemical precipitation was improved. Fibrin gel treatment experiment was used to evaluate the application of hydrogel combined with nanotechnology in HCC intraoperative bleeding. The study results showed that nanocomposite gel could effectively reduce the intraoperative bleeding and postoperative recurrence of HCC, and greatly increase the safety of surgical intervention.

### 4.2 Application in ablation

Thermal ablation is a common intervention for HCC in addition to surgery, which can greatly control local tumor growth and prolong the long-term survival of HCC patients (Kang and Rhim, 2015). Ablation removes damaged cells and tissues by applying electromagnetic waves and high heat. Radiofrequency ablation (RFA) and microwave ablation (MWA) are the most widely used ablation procedures in clinical practice (Deng et al., 2023). Compared with surgery, ablation has a wider range of symptoms and a lower mortality rate (Kim et al., 2014). However, the selection of appropriate methods to transfer heat to the tumor site is currently the top priority in the treatment of thermal ablation. Nanoparticles can focus energy from external sources on the tumor site, thereby inducing local tumor destruction and minimizing adverse effects on other tissues (Beik et al., 2016).

Wu S. et al. (2020) used Disaturated-phosphatidylcholine (DSPC), 1,2 distearoyl-snglycero-3-phosphoethanolamine-N-[methoxy (poly-ethyleneglycol)-2000] (DSPE-PEG2000), Cholesterol mixture, DNPs was prepared by encapsulating doxorubicin (DOX) in liposomes by remote drug loading method. Flow cytometry was used to detect the uptake of DNPs by HCC cells. The results indicated that DOX uptake via DNPs was 1.5 times higher compared to free DOX. At the same concentration, the cell viability of DNPs was much lower than that of DOX (41.64% ± 8.88%vs52.77% ± 3.28%). Mild microwave ablation (MWA) combined with DNPs can significantly improve the ablation efficiency of HCC and significantly inhibit liver tumors in mice, and the survival rate of mice in the MWA + DNPs group was 100% on the 14th day.

To enhance the therapeutic efficacy against HCC, Jin Y. et al. (2018) developed nanoparticles composed of SP94-modified polypyrrole (PPy),bovine serum albumin (BSA), and ICG for localized treatment of solid tumors. To assess the photothermal decomposition effects of these SP94-modified PPy-BSA-ICG nanoparticles, near-infrared light irradiation was applied separately to PBS and the nanoparticles. The results indicated that PBS exhibited negligible temperature changes under irradiation, whereas the temperature of the SP94-modified PPy-BSA-ICG nanoparticles increased in correlation with their concentration. *In vivo* experiments involved direct intratumoral injection of PBS or SP94-modified PPy-BSA-ICG nanoparticles at a dose of 10 mg/kg in Hep3B tumor-bearing mice. The study found a significant reduction in tumor volume in the PPy-BSA-ICG plus laser group, and complete tumor ablation in the SP94-modified PPy-BSA-ICG plus laser group. In conclusion, these nanoparticles have been successfully utilized in image-guided photothermal therapy for HCC, providing effective and precise ablation treatment. To overcome the heating effect of low efficiency of radiofrequency ablation, Somasundaram et al. (2016) used ion crosslinking technology to prepare DOX-loaded tin ion doped alginate NPs, and reacted the crosslinking agent solution with polyvinyl imide to prepare ADNPs containing alginate -DOX. GAGNPs was obtained by the glycosylation of ADNPs.The toxic effects of GAGNPs on HEPG hepatocellular carcinoma cells and RF thermal response *in vitro* were subsequently evaluated. The study results indicated that the cell mortality rate for the GAGNPs + RFA treatment group was significantly higher (81%) compared to the group treated with RFA alone (52.5%). Furthermore, it has been demonstrated that GAGNPs can enhance the thermal response of RF *in vitro*. NPs was labeled with Techentium-99m to optimize liver localization in SD rats. The study results show that GAGNPs not only enhances RF hyperthermia in HCC rat models, but also accurately localizes liver tumors for uniform enhancement of ablation.

### 4.3 Transcatheter arterial embolization

The blood in normal hepatic parenchyma is supplied by 75% venous blood and the remaining 25% by hepatic artery. However, the abnormal formation of new blood vessels in the process of liver cancer makes liver malignant tumors preferentially obtain the blood supply of liver arteries, This is the basis of TACE for the treatment of HCC. Compared with systemic chemotherapy, the application of TACE not only delivers high doses of cytotoxic drugs to the tumor, increases the local tumor drug concentration, and induces tumor ischemia and hypoxic necrosis in conjunction with arterial embolization, but also greatly reduces systemic adverse effects in patients (Wáng et al., 2015; Yuan et al., 2023a). However, in clinical practice, for unresectable HCC tumors with a diameter of more than 10 cm, the course of TACE may cause various adverse reactions such as iodized oil ectopic embolism, fever, abdominal pain, and liver abscess (Guo et al., 2023). Among TACE-induced ectopic embolism, cerebral embolism and pulmonary embolism have been reported in several articles (Jia et al., 2012). The underlying mechanism for this occurrence remains unclear and may be related to hepatic arteriovenous shunt with hepatic vein invasion in hepatocellular carcinoma, the liquid sulfurized oil its particle size is unstable, and the oil bolus enters the body circulation through the pulmonary arteriovenous shunt (Zhao et al., 2008; Wu et al., 2023). In TACE, it has been found in many years of clinical practice that iodized oil is cleared too fast in some patients, which can not achieve the purpose of long term embolization. In addition, due to the fast release of drugs in iodized oil, the drugs not only can not maintain a high concentration in tumor tissues for a long period of time, but also the fast release of the high concentration of drugs into the circulatory system causing a variety of acute side effects (Peng et al., 2022). Based on the above, some patients need to undergo multiple interventions, which tend to be more burdensome to the patient’s liver function. In addition, which is also the theoretical basis of TACE treatment (Kishore et al., 2020). Therefore, selecting appropriate embolic agents is crucial for the success of TACE treatment (Osuga et al., 2012; Jia et al., 2022).

In recent years, researchers have continued to focus on the functionalization of nanomaterials, opening up new possibilities for nanomedicine to contribute to overcoming the current challenges of TACE therapy (Ladju et al., 2022). The larger surface area of nanomaterials makes their surface reaction activity and reactivity significantly improved, which not only reduces the toxic side effects of drugs, but also realizes the precise control of drug release. Secondly, nanomaterials show strong advantages in mechanics, hardness, abrasion resistance and toughness. Compared with traditional iodized oil, nanoformulations are more stable as embolic agents while reducing damage to normal tissues. It is clear that the widespread use of a wide range of nanomaterials in TACE provides new options for trials to treat HCC, as well as strong support for the development of novel functional nanoplatforms.

In their 2018 study, Zeng et al. (2018) employed the electrospray method to create calcium alginate microspheres embedded with tantalum nanoparticles (Ta@CaAlg). Doxorubicin (DOX) was chosen as a model drug to evaluate the transport capacity and release characteristics of Ta@CaAlg, using X-ray imaging and computed tomography (CT) for assessment. The results demonstrated that Ta@CaAlg microspheres possess both embolic and contrast agent functionalities, highlighting their significant potential for clinical use in TACE.

The development of HCC is influenced by the establishment of a local tumor microenvironment, with hypoxia playing a crucial role in the formation of this HCC microenvironment. After TACE treatment, local hypoxia induced residual tumor cell angiogenesis and metastasis (Gai et al., 2020). Therefore, the regulation of the tumor microenvironment has become the focus of HCC treatment. Cellular autophagy has been found to be an important feature of TME formation in solid tumors. Under the tumor microenvironment of hypoxia and ischemia, the autophagy program of HCC cells is activated, and it is also capable of causing resistance to TACE chemotherapy, Therefore, the use of autophagy inhibitors is thought to improve the therapeutic efficacy of TACE (Li et al., 2024). Based on this Yuan et al. (2023b) developed and synthesized a ph-responsive polyacrylic acid/calcium phosphate nanoparticles (PAA/CaP NPs). and used it as a carrier for epirubicin (EPI), PAA/CaP NPs exhibited significant autophagy inhibitory activity. In a rabbit VX2 *in situ* liver tumor model, researchers integrated CaP-EPI into a lipol-based chemoembolization system for the *in vivo* treatment of hepatocellular carcinoma. The findings indicated that PAA/CaP nanoparticles not only enhanced the therapeutic efficacy of TACE by inhibiting autophagic flux but also significantly minimized various side effects associated with TACE.

Su (2021) for exploring the value of TACE combined with sorafenib (SFB) nanocarrier system (AB-SFB-NP) in the treatment of HCC. Forty-two postoperative patients with hepatocellular carcinoma were collected clinically and divided into a control group and an experimental group according to the patients’ wishes. After 3 months of intervention respectively, the patients’ blood routine, liver function, enhanced CT and MRI results were collected and statistically analyzed, and the results showed that the DCR of the experimental group was 88.9%, and the DCR of the control group was 58.4%.Subsequently, the adverse reactions of the two treatment groups were assessed, and the hand-foot syndrome, diarrhea, and bone marrow suppression were lower in the experimental group than in the control group. It demonstrated that SFB combined with AB-SFB-NP nanodelivery had a higher safety profile.

Integrins constitute a substantial family of transmembrane cell adhesion molecules that are critical for mediating adhesion among cells or between cells and the extracellular matrix (ECM) (Cai and Chen, 2006). Qian et al. (2016) divided HCC rat models into TACE + GRGDSP (Gly-Arg-Gly-Asp-Ser-Pro), integrin inhibiter-loaded nanoparticle group, TACE + GRGDSP group, and TACE alone group. MRI imaging was used to determine tumor size the day before interventional surgery. Then laparotomy was performed. The results indicated that TACE combined with GRGDSP-loaded nanoparticles significantly reduced tumor size during the experimental observation period. Immunohistochemistry revealed that the expression of matrix metalloproteinase-9 (MMP-9) and vascular endothelial growth factor (VEGF) in hepatocellular carcinoma was lower in the TACE + GRGDSP nanoparticle group compared to the control group. This suggests that using TACE + GRGDSP-loaded nanoparticles in HCC can significantly delay tumor growth and intrahepatic metastasis compared to TACE alone or TACE with integrin inhibitors.

Based on previous findings, nanoscale hydroxyapatite (nHAP) demonstrated good hepatocyte compatibility, safety, and tumor cell-specific inhibition (Fu et al., 2005). Therefore, Li et al. (2012) tried to use nHAP as a gene carrier for targeted transarterial embolization gene therapy for liver cancer. We applied surface-modified nHAP and p53 expression plasmodies formed from polypolex to HePG2 cells *in vitro*, and then transfected and embolized liver tumors with iodized oil/NHAp-PLL-p53 emulsion via arterial infusion. The results indicated that nanohydroxyapatite (nHAP) exhibited superior dispersibility and targeted tumor effects compared to polyplexes. *In vitro* experiments demonstrated that nHAP ensured the safety of normal liver cells while maximizing the induction of apoptosis in tumor cells, highlighting its potential in targeted cancer therapy.

### 4.4 Nano drug delivery system

For patients undergoing surgical resection, liver transplantation, or those with local TACE treatment failure. Systemic therapy with small molecule drug targeting is widely used. However, the systemic chemotherapy of HCC has the disadvantages of dose-limited cytotoxicity and easy drug resistance, and the therapeutic effect is not satisfactory (Dutta and Mahato, 2017). Nanoparticles, as drug delivery carriers, improve pharmacokinetics, biological distribution, accumulation of cytotoxic drugs at tumor sites, and precise targeting, enhancing local therapeutic effects while minimizing the side effects of systemic therapy (Sharma et al., 2017). Therefore, the construction of drug loading and delivery systems for HCC therapy has become the focus of current research (Lu et al., 2024) (Table 2).

**TABLE 2:** Application of nano drug delivery in hepatocellular carcinoma.

Medicine	Nanomedicine	Average particle size (nm±SD)	Zata potential (mv ± S D)	EE%	LE%	Cumulative release%	References
SRF GA	SRF and GA-loaded NLCs	29.28	Not tested	93.1	14.21	84	Wang et al. (2021a)
GA	GA-NLCs	156	−4.99 ± 1.3	86.3 ± 1.5	12.2 ± 2.11	Not tested	Rahman et al. (2019)
DOX SOR	NAcGal-DOX/SOR LNPs	121.2 ± 3.5	−37.4 ± 3.6	DOX (80.7 ± 2.9) SOR (83.2 ± 3.3)	DOX (5.6 ± 0.5) SOR (4.1 ± 0.4)	Not tested	Duan and Liu (2018)
glutathione	Ag-S	20–50	−8 to 10	Not tested	Not tested	Not tested	Thai et al. (2021)
CUR BBR	CUR&BBR/GA-HA-Lip	159.39 ± 3.16	−0.24 ± 0.35	CUR (93.66 ± 3.08) BBR(92.59 ± 5.45)	CUR (2.15 ± 0.07) BBR(2.13 ± 0.13)	CUR 81.09 BBR 81.93	(Wu et al., 2022)
LUT	LUT-ENPs	267 ± 8.6	−30.1 ± 1.2	Not tested	Not tested	84.47 ± 1.37	Elsayed et al. (2021)
RT	RT-PLGA-NP	211	−21.3–22.8	77.83 ± 1.77	6.39 ± 0.16	71	Pandey et al. (2018)
SOR	SPVNs	215.70 ± 0.36	−26.01 ± 0.65	90.35	Not tested	73.81 ± 4.53	Tang et al. (2022)
Paclitaxel	P-NPs	118.47 ± 52.21	−41.6	80	3.81	Not tested	Jin et al. (2018a)
CNB	CNB-PLGA-PSar-NPs	192.0 ± 3.67	−24.18 ± 3.34	76.35 ± 2.76	14.67 ± 3.86	84.25 ± 7.89	Bhattacharya et al. (2023)
SOR	aPD-1-PLTM@HMSNs@Sora	1,069.33 ± 41.86	−8.46 ± 2.41	52.99 ± 1.90	20.94 ± 0.6	Not tested	Da et al. (2024)
5-FU	NEM-5-FU-LP	191.4 ± 14.76	Not tested	Not tested	30.71 ± 2.58	92.5 ± 3.23	AlQahtani et al. (2019)
Curcumin Paclitaxel	CU-PTX-LNP	Not tested	No test	>90%	3.39 ± 0.30	Cu:60 Ptx:90	Wei et al. (2023)
MX	MX-LPG	105.57 ± 0.75	−29.28 ± 1.73	97.33 ± 0.37	1.37 ± 0.10	Not tested	Huang et al. (2012)
RX	RX-HA-CS NP	208.7 ± 4.7	−29.1 ± 4.5	92	Not tested	Not tested	Almutairi et al. (2019)
Boswellic acids, curcumin and naringin	Boswellic acids NPs curcumin NPs naringenin NPs	277.50 ± 29.23	−31.23 ± 4.54	Not tested	Not tested	Not tested	Elnawasany et al. (2023)
Dox	(DOX/MSN-CPN)	125.01 ± 1.52	−20.20 ± 0.45	Not tested	10.55 ± 0.15	>80	Liu et al. (2023)
UA EGCG	UA@EGCG-Apt NPs	160.0	−26.1	85.2	Not tested	70	Zhang et al. (2021a)
Dox FA	FA-EYLNs-Dox	48	−45	75.33 ± 8.08	Not tested	Not tested	Tang et al. (2020b)
CDF	CDF/G4-PAMAM-GAL	48.7 ± 7.03	−8.3 ± 1.24	75.5 ± 12	17.7 ± 5.43	Not tested	Yousef et al. (2018)
Fe3O4-PEI@HA-RSL3	Fe3O4-PEI@HA-RSL3	10–16	−24.8 ± 3.43	53.48	26.74	78.45	Liang et al. (2023)
Apocynin	APO-loaded GC-coated PLGA NPs	224.29 ± 3.27	12.07 ± 0.67	34.22 ± 1.02	Not tested	59.93 ± 0.13	Anter et al. (2023)
DOC FA	DOX@UiO-68-FA	140 ± 21	−13.5 ± 0.4	No test	4.79wt	Not tested	Li et al. (2016)

The FOLFOX regimen, which includes folinic acid (FnA), fluorouracil (5-Fu), and oxaliplatin (OxP), is a common standard chemotherapy treatment for patients with hepatocellular carcinoma (Lyu et al., 2018). In a study, Cheng et al. developed nano-liposomes co-loaded with cisplatin (CDDP) and curcumin (CUR). The MTT assay was utilized to evaluate the cytotoxicity of different formulations on HepG2 cells *in vitro*. The findings indicated that cisplatin nano-liposomes (CDDP-Lip) exhibited significantly higher cytotoxic activity against HepG2 cells than cisplatin solution (CDDP-Sol) at equivalent concentrations. Additionally, the anti-tumor efficacy of these formulations was assessed using a HepG2 xenograft tumor model. Among the treatment groups, mice treated with CDDP/CUR-Lip exhibited the smallest tumors and the most pronounced anti-tumor effect (Cheng et al., 2018). In another *in vitro* study, CDDP and Farnesol (FAR) were co-encapsulated in polylactic acid-glycolic acid copolymer nanoparticles (NCDDPFAR), and the researchers found that NCDDPFAR treatment accelerated drug fluidity compared to monotherapy. Prolonged drug release (60–78% vs 80%–96.45%) and showed the highest apoptotic cell death compared to the other treatment groups (Mondal and Khuda-Bukhsh, 2020). In addition, studies have shown that the use of nuclear localization sequentially modified metal-organic frameworks supported by CDDP and oxidized nitro domain protein 1 (NOR1) shRNA can inhibit NOR1 expression to reverse chemotherapy resistance and exert anti-tumor effects, providing an effective gene therapy for HCC cisplatin resistance (Huang et al., 2023).

Sorafenib (SFN) is an oral multi-targeted therapy employed for the treatment of unresectable or metastatic hepatocellular carcinoma, leading to an increase in the median overall survival of patients with HCC. However, its clinical efficacy is limited due to poor water solubility and slow dissolution, which hinder gastrointestinal absorption. Consequently, only about 30% of patients benefit from sorafenib, and resistance typically develops within 6 months (Elsayed et al., 2019; Tang W. et al., 2020). To address the resistance and therapeutic limitations of HCC, researchers have developed various new SFN nanocarriers. These innovations aim to enhance drug delivery, improve solubility, and increase bioavailability, thereby improving the overall efficacy of SFN in treating HCC (Kong et al., 2021).

In recent years, chitosan (CS) has garnered significant attention for its exceptional biological properties. Notably, chitosan nanoparticles (CS NPs) have been utilized to support SFN, resulting in the development of SFN-CS NPs, which show promise for HCC treatment. Cytotoxicity assays on HepG2 cells revealed that free sorafenib required a higher dose to achieve 50% cytotoxicity, whereas SFN-CS NPs achieved the same cytotoxic effect at a lower dose (Albalawi et al., 2023). In another study, researchers successfully prepared the drug-loaded CNTs (CNT-SFN) microcapsules by loading sorafenib onto functionalized carbon nanotubes via physical adsorption. The therapeutic effect of CNT-SFN in HCC was subsequently evaluated *in vitro* and *in vivo*. *In vitro* results showed that HepG2 cells treated with CNT-SFN had significantly reduced viability compared with free sorafenib, with IC50 values of 10.24 μmol/L and 4.05 μmol/L, respectively. *In vivo* results also indicated that CNT-SFN was superior to conventional SFN in systemic toxicity and tolerance. To overcome the drawback of SFN resistance in HCC, Xu et al. prepared hollow mesoporous manganese dioxide (H-MnO2) sorafenib and chloroprotein e6 (Ce6), and used dopamine-modified nanoparticles to optimize biocompatibility. Finally, MCS NPs was prepared. It was found that MCS NPs could reverse sorafenib resistance by improving the hypoxia of TME and down-regulating the expression of HIF-1α. Moreover, it can directly kill tumor cells or inhibit tumor progression by disrupting tumor blood vessels (Xu et al., 2024). In conclusion, constructing multifunctional nanoplatforms can significantly improve the therapeutic efficiency of SFN, offering a novel approach to overcoming SFN resistance in HCC treatment.

In recent years, natural compounds have been widely used in daily life as drugs or health products because of their multi-target effects and high level of safety (Zhao et al., 2020). Increasing evidence indicates that natural products can inhibit tumor cell proliferation by inducing autophagy and depriving liver cancer cells of essential energy sources. These findings suggest a promising avenue for developing novel therapeutic strategies against HCC. The production of these effects has also been shown to be closely related to PI3K/AKT, MAPK, AMPK, Wnt/β-catenin, Beclin-1, and iron autophagy (Hu et al., 2013; Chen et al., 2024). The application of nanotechnology combines the methods of embedding, encapsulation and adsorption of the active ingredients of natural products with nanocoliths, which can directly bring the active ingredients of natural polysaccharides directly to tumors and lymphatic organs and enhance immune response (Schwendener, 2014; Liu et al., 2018).

Guo et al. (2021) prepared Cur-loaded small molecule glycyrrhetic acid (GA) -Angelica polysaccharide APs-Disulfide bond (DTA)-Cur nanomicelle by dialysis method (GACS-Cur), and encapsulated GACS-Cur (GACS-Cur@RBCm) with erythrocyte membrane to prolong the circulation effect. The tumor growth was assessed in a nude mouse model carrying cancer cells, revealing that GACS-Cur@RBCm exhibited superior tumor tissue targeting and enhanced tumor inhibition. This suggests that APS nanoparticles can increase drug concentration at the tumor site, thereby improving the efficacy of anti-tumor drugs. Additionally, immunomodulatory effects showed that the GACS-Cur@RBCm intervention group had 1.9 times higher expression of IL-12, TNF-α, IFN-γ, and CD8^+^ T cell infiltration compared to the saline group.

Ber-loaded silver nanoparticles (Ber-AgNPs) and Ber-loaded selenium nanoparticles (Ber-SeNPs) synthesized by Khaled et al., (2024). After incubation of Ber-AgNPs or Ber-SeNPs with IC50 at 3/1 and 2/1, a decrease in the level of anti-apoptotic protein (Bcl-2) was observed, accompanied by an increase in the level of pro-apoptotic protein (Bax), compared with the control group. In addition, P53 and caspase-3 protein levels were significantly increased by 89% and 97% in HepG2 cells treated with higher doses of the two Ber-NPs.In addition, P53 and caspase-3 protein levels were significantly increased by 89% and 97% in HepG2 cells treated with higher doses of the two Ber-NPs.

Xu et al. (2020) by urocanic acid and α-lipoic acid (α-LA) to obtain a copolymer (LA-URPA), encapsulated ginsenoside Rh2 with LA-URPA. These nanoparticles exhibited a high encapsulation efficiency of 86.00% and demonstrated a significant dual response under acidic conditions. Additionally, Rh2 NPs with this dual pH/reduction responsiveness displayed enhanced cytotoxicity compared to free Rh2 after being incubated with HepG2 cells for 72 h, with drug release continuing beyond 96 h.

### 4.5 Sensitizer for radiotherapy

As clinical applications have expanded, radiotherapy has emerged as a crucial component of the multidisciplinary approach to treating HCC (Lewis et al., 2022). However, the tumor specificity of radiotherapy is poor, and the highly penetrating photons tend to damage the normal liver tissue around the tumor (Kalogeridi et al., 2015). Nanoparticles have been widely used in the treatment of solid tumors because of their high permeability and retention effect (Rancoule et al., 2016). It has been suggested that the use of nanoparticles as radiosensitizers broadens the prospects for radiation therapy (Boateng and Ngwa, 2019). This allows radiotherapy to precisely eliminate liver malignancies without damaging the liver and other cells (Pérez-Romasanta et al., 2021). Currently, there is significant research interest in nano-sensitizers for radiotherapy, especially those based on precious metals, rare earth metals, and semiconductor metals (Song X. et al., 2023). Chen Y. et al., (2023) prepared albumin-modified GNPs (Alb-GNPs) and observed the radiation sensitization and biotoxicity of the particle in a tumor-bearing mouse model. At the radiation dose of 6 Gy, the tumor volume increased the most slowly in the Alb-GNPs + X-ray group, and the clone formation experiment showed that the sensitization enhancement ratio (SER) was as high as 1.432 in the experimental group. Much higher than the pure X-ray group. In addition, a variety of Rare earth metal-based nano-radiosensitizers are important for improving radiation therapy and enhancing radiotherapy sensitivity. Verry et al., (2019) created a gadolinium-based nanoparticle known as AGuIX, which has recently shown its efficacy as both a theranostic and radiosensitizing agent in various studies. The results of *in vitro* studies showed that the application of AGuIX increased the radiation efficiency by 1.1–2.5 times. In addition, AGuIX was shown to improve sensitivity to radiation therapy in 6 animal tumor models. As an approach to counteract tumor hypoxia and the associated immunosuppressive microenvironment, Liu et al. (2021) developed an innovative theranostic agent based on Bi/Se NPs. By loading these NPs with Lenvatinib (Len), they formulated Bi/Se-Len NPs, which were utilized for *in vivo* CT image-guided stereotactic body radiation therapy (SBRT) sensitization in mice models. The *in vivo* studies demonstrated that Bi/Se-Len NPs could accurately delineate the radiation therapy (RT) region. Furthermore, by alleviating hypoxia at the tumor site, these nanoparticles enhanced the infiltration of tumor-infiltrating CD4 and CD8 T lymphocytes around the tumor. Cheng et al. (2017) also proved that Bi2S3 nanorods-mediated RT could not only reduce the hypoxia in the tumor region by inhibiting the expression of HIF-1, but also enhance the therapeutic efficacy of RT by decreasing the activities of the DNA repair enzymes Poly (ADP-ribose) polymerase and Rad 51, and inducing DNAs to kill tumor cells.

Zheng et al. (2013) tested the effect of nanoparticles combined with radiotherapy on HCC, and chose nano-gold (GNPs) and nano-silver (SNPs) as the starting point to search for particles that enhance the radiotherapy effect. The results indicated that the use of nano-gold and nano-silver not only decreased the viability of HepG2 cells but also significantly increased their radiosensitivity. In another study, Guo et al. (2017) synthesized 14.4 nm and 30.5 nm polyethylene glycol (PEG) coated (GNPs) by chemical reduction reaction, and used clonal cell survival tests to determine the radiosensitization effects of two nanoparticles of different sizes in H22 and HepG2 cells, respectively. The survival rate of H22 and HepG2 cells in the 14.4 nm group was significantly lower than that in the control and 30.5 nm groups. This indicates that the application of gold nanoparticles (GNPs) in in vitro radiation therapy significantly enhanced the irradiation effect on these liver cancer cells.

Radioresistance induced by anoxic microenvironment is one of the main obstacles to clinical radiotherapy of HCC (Kabakov and Yakimova, 2021; Zeng et al., 2021). The continuous development of nanomedicine provides the possibility to alleviate the hypoxic microenvironment of solid tumors. Researchers have developed oxygen microcapsules stabilized by polydopamine nanoparticles. These microcapsules can rapidly improve the hypoxic microenvironment in HCC and maintain elevated oxygen levels for an extended period. In HCC mouse models, local injection of these oxygen microcapsules has been shown to significantly enhance the efficacy of radiotherapy (Dai et al., 2021).

In another study, Ruoling Gao and colleagues developed curcumin-hemoglobin nanoparticles (Cur@Hb) Drug release in a simulated *in vitro* tumor microenvironment showed that more than 60% of curcumin was released from Cur@Hb at 72 h. Subsequently, under the same radiation conditions, the radiosensitization effect of nanoparticles on SMMC7721 cells was demonstrated by cloning survival experiments. The results showed that the radiosensitization ratios of SMMC7721 cells treated with Hb, Cur and Cur@Hb were 1.030, 1.231 and 1.236, and 1.328, 1.318 and 1.510, respectively, under normal oxygen and hypoxia conditions. These results indicate that Cur@Hb can enhance the radiosensitivity of SMMC7721 cells under both normal and hypoxia conditions (Gao et al., 2022).

### 4.6 Nano-vaccines

Cancer immunotherapy relies on the patient’s own immune system to activate an adaptive anti-tumor response to fight cancer (Saleh and Shojaosadati, 2016). Among the various forms of immunotherapy, cancer vaccines have demonstrated their ability to induce tumor-specific immunity through mechanisms such as the release of antigen, the uptake of antigen by antigen presenting cells, and the activation and activation of T cells (Topalian et al., 2011). Compared with traditional vaccines, nano-vaccines use nanomaterials as carriers to deliver specific antigens and adjuvants, which has unique advantages in tumor immunotherapy targeting therapy, prolonging drug cycle, identifying specific targets, reducing drug toxicity, etc., so as to achieve better treatment or prevention of tumors (Weissleder et al., 2005; Kalaydina et al., 2018; Zhang D. et al., 2020; Oroojalian et al., 2021; Saravanakumar et al., 2022).

Wang Y. et al. (2022) developed a neoantigen nanovaccine utilizing acid/photosensitive dendritic cells. Flow cytometry analysis indicated that the mD@cSMN nano-vaccine significantly boosted T cell activation. *In vitro* studies revealed that after 36 h of co-incubation with T cells treated by mD@cSMN, the rates of apoptosis and necrosis in tumor cells rose to 51.6%, which was notably higher than that observed in the control group. To assess the immune stimulation effects of the mD@cSMN nano-vaccine *in vivo*, an ELISpot assay was performed following subcutaneous injection of the nano-vaccine into BALB/c mice over a period of 14 days. The results showed that mD@cSMN nano-vaccine more effectively enhanced IFN-γ secretion compared to other treatments. These findings indicate that the mD@cSMN nano-vaccine can directly stimulate T cell activation and proliferation, leading to effective destruction of H22 hepatoma cells.

Zhang L. et al. (2021) created a self-assembling, vehicle-free multi-component antitumor nano-vaccine (SVMAV). This formulation was made using an unsaturated fatty acid, docosahexaenoic acid (DHA)-conjugated antigen, and R848, which acts as a Toll-like receptor 7/8 agonist to encapsulate stattic, an inhibitor of signal transducer and activator of transcription 3 (STAT3). Researchers then established a general platform for neoantigen-targeted personalized cancer vaccines known as HLS@SVMAV to evaluate its clinical efficacy. Hepa1-6 cells were injected into the left liver lobe to create a mouse model of *in-situ* hepatoma. The mice received either HLS@SVMAV or anti-PD-1 therapy. Results indicated that the liver cancer model exhibited significant resistance to anti-PD-1 treatment; however, tumors in mice treated with HLS@SVMAV showed considerable shrinkage. Furthermore, while anti-PD-1 therapy decreased the population of F4/80CD86+M1 macrophages, HLS@SVMAV did not have a notable impact on M1 macrophage levels. These results imply that SVMAV-targeting nanovaccines could represent an innovative approach for treating HCC.

Chen et al. (2018) incorporated one Toll-like receptor (TLR)-7/8 agonist CL097 as with mannosylated liposomes (LPMan) adjuvant was prepared to contain Glypican-3 (GPC3) nanovaccines containing CL097 (LPMan-GPC3/CL097). The study results show that LPMan-GPC3/CL097promotes migratory DC antigen uptake, maturation and migration to draining lymph nodes. The authors injected DEN into mice after birth to induce hepatocyte damage and simulate the formation of HCC. GPC3 overexpression of premalignant hepatocyte clusters was found at 8 weeks of DEN injection, followed by 4 immunizations of HBV transgenic mice treated with DEN using LPMan-GPC3/CL097 every 2 weeks. By week 15, liver tumor nodules were found in all of the non-immunized and pseudo-immunized mice, while small tumor nodules were found in only one-fifth of the mice in the experimental group. The results clearly indicate that the application of LPMan-GPC3/CL097 can prevent the development of HCC in the presence of cancerous nodules in cirrhosis.

## 5 Summary and prospect

In recent years, nanomaterials have significantly contributed to advancements in cancer treatment, particularly by improving drug bioavailability through enhanced pharmacokinetic and pharmacodynamic characteristics. In contrast to low-molecular-weight immunomodulators, nanoscale small molecule drugs provide controllable pharmacokinetic profiles and can boost immune activation through synergistic effects. This advantage is due to their unique size and capacity to co-load various functional domains. Nanotechnology also offers ways to allow chemotherapy to directly and selectively target cancer cells, guide surgical removal of tumors, and improve treatment outcomes based on radiation and other current treatment modalities.

These can reduce the risk of toxicity for patients and improve the likelihood of prolonged survival. The application of nano-vaccines has also continuously broadened the means of tumor treatment, which may become an effective means to overcome solid tumors, and has a wide range of application potential in tumor treatment. Despite the widespread interest in the use of nanomaterials in various tumor therapies, their clinical translation still faces serious challenges. As with many clinical therapeutic options, nanomaterials are not entirely non-toxic, and their toxicity and biological activity pose potential threats to the human body. The distribution, *in vivo* toxicity, behavioral pathways and metabolic pathways of some nanomaterials in human tissues and organs are still areas that scientists are currently investigating. while nanomaterials are able to cross the blood-brain barrier while treating a variety of neurological disorders, they can also produce a variety of neurotoxicity (Nel et al., 2006). In addition, the preparation and storage of nanomaterials face a number of challenges, and nanomedicines need to maintain an ultra-stable structure in order to have the desired effect. Therefore, there is still a long way to go before the preparation of nanoformulations can move from the laboratory to industrial production (Wu L. P. et al., 2020). Current research areas on nano-formulations are mainly focused in cell lines, cell spheres and implantation models, which do not accurately simulate the human tumor microenvironment, thus affecting the clinical evaluation and application of nanomedicines. Finally, the application of nanotechnology in tumors also needs to overcome the immunosuppression caused by multiple mechanisms. All of the above are factors that constrain the widespread application of nano-agents. However, despite the strong interest in the application of nanomaterials in various tumor therapies, their clinical conversion still faces serious challenges. Like many clinical treatment options, Nanomaterials are not completely non-toxic, and their toxicity and biological activity pose potential threats to the human body.

The distribution of some nanomaterials in tissues and organs in the human body, *in vivo* toxicity, behavioral pathways and metabolic pathways are still the fields that scientists are currently studying. In clinical applications of nanomaterials, researchers should improve drug delivery methods to provide safer and more reliable therapeutic derivatives by optimizing the interaction between the physical and chemical properties of nanomaterials. Therefore, in the future strategy of tumor treatment, researchers can try to apply different materials in combination to improve the targeting of drugs. Researchers can also change the shape and size of the nanomaterials to improve the permeability and targeting ability of the nanomaterials, so as to better exert the anti-tumor effect. HCC is a complex and varied disease, and it is often difficult to control the progression of the disease with a single therapy, so in the future, we need to actively explore more types of nanomaterials throughout a variety of therapeutic options in order to achieve better therapeutic effects, and bring new hope to HCC patients. In the future, we should more actively explore more types of nanomedicines across multiple therapeutic regimens to achieve better therapeutic effects and bring new hope to HCC patient.

## Glossary

HCC: Hepatocellular carcinoma
HBV: Hepatitis B virus
HCV: Hepatitis C virus
NAFLD: non-alcoholic fatty liver disease
NASH: non-alcoholic steatohepatitis
ALD: alcoholic liver disease
TLR4: Toll-like receptor
BCLC: Barcelona Clinic Liver Cancer
TACE: transcatheter chemoembolization
BBB: Blood-Brain Barrier
CNS: central nervous system
NPs: Nanoparticles
TAM: tumor-associated macrophages
MDSC: bone marrow-derived suppressor cells
TAN: tumor-associated neutrophils
CAF: cancer-associated fibroblasts
Tregs: regulatory T cells
IL-34: interleukin34
IL-12: interleukin 12
IL-10: interleukin 10
DCs: dendritic cells
ROS: reactive oxygen species
Pmhc: peptide-MHC
BMP2: bone morphogenetic protein 2
LSAMP: limb-system associated membrane protein
CYLD: CYLD lysine 63 deubiquitinase
EGF: epidermal growth factor
PDGF: platelet-derived growth factor
EMT: epithelial mesenchymal transformation
TCN: twin-like core-shell nanoparticles
TIDC: tumor-infiltrating dendritic cells
CIB: immune checkpoint-blocking
FTO: fat mass and obesity associated gene
ECM: extracellular matrix
TGF-β: faction-β1
NIR: near-infrared fluorescence
ICG: Indocyanine green
IGS: image-guided surgery
SHIFT: super-stable homogeneous intermixed formulation technology
PEG: polyethylene glycol
RFA: Radiofrequency ablation
MWA: microwave ablation
DSPC: Disaturated-phosphatidylcholine
MWA: Mild microwave ablation
PPy: polypyrrole
BSA: bovine serum albumin
CT: computed tomography
GRGDSP: Gly-Arg-Gly-Asp-Ser-Pro
MMP-9: matrix metalloprotein-9
VEGF: vascular endothelial growth factor
nHAP: nanoscale hydroxyapatite
FnA: folinic acid
5-Fu: fluorouracil
OxP: oxaliplatin
CDDP: cisplatin
CUR: curcumin
CDDP-Lip: cisplatin nano-liposome
CDDP-Sol: sole cisplatin
FAR: Farnesol
NOR1: nitro domain protein 1
SFN: Sorafenib
CS: chitosan
CS NPs: chitosan nanoparticles
SFN-CS NPs: sorafenib supported by chitosan nanoparticles
**H-MnO**_**2**_: hollow mesoporous manganese dioxide
GA: glycyrrhetic acid
AP: Angelica polysaccharide
Ber-AgNPs: Ber-loaded silver nanoparticles
Ber-SeNPs: Ber-loaded selenium nanoparticles
α-LA: α-lipoic acid
GNPs: nano-gold
SNPs: nano-silver
PEG: polyethylene glycol
Cur@Hb: curcumin-hemoglobin nanoparticle
NLCs: nano lipidic carriers

## References

[B1] Albalawi F. Hussein M. Z. Fakurazi S. Masarudin M. J. (2023). Fabrication and characterization of nanodelivery platform based on chitosan to improve the anticancer outcome of sorafenib in hepatocellular carcinoma. Sci. Rep. 13 (1), 12180. 10.1038/s41598-023-38054-4 37500670 PMC10374537

[B2] Almutairi F. M. Abd-Rabou A. A. Mohamed M. S. (2019). Raloxifene-encapsulated hyaluronic acid-decorated chitosan nanoparticles selectively induce apoptosis in lung cancer cells. Bioorg Med. Chem. 27 (8), 1629–1638. 10.1016/j.bmc.2019.03.004 30879864

[B3] AlQahtani S. A. Harisa G. I. Badran M. M. AlGhamdi K. M. Kumar A. Salem-Bekhit M. M. (2019). Nano-erythrocyte membrane-chaperoned 5-fluorouracil liposomes as biomimetic delivery platforms to target hepatocellular carcinoma cell lines. Artif. Cells Nanomed Biotechnol. 47 (1), 989–996. 10.1080/21691401.2019.1577887 30873877

[B4] Anter H. M. Aman R. M. Othman D. I. A. Elamin K. M. Hashim I. I. A. Meshali M. M. (2023). Apocynin-loaded PLGA nanomedicine tailored with galactosylated chitosan intrigue asialoglycoprotein receptor in hepatic carcinoma: prospective targeted therapy. Int. J. Pharm. 631, 122536. 10.1016/j.ijpharm.2022.122536 36572262

[B5] Anwanwan D. Singh S. K. Singh S. Saikam V. Singh R. (2020). Challenges in liver cancer and possible treatment approaches. Biochim. Biophys. Acta Rev. Cancer 1873 (1), 188314. 10.1016/j.bbcan.2019.188314 31682895 PMC6981221

[B6] Arbab A. S. Ichikawa T. Araki T. Toyama K. Nambu A. Ohsawa S. (2000). Detection of hepatocellular carcinoma and its metastases with various pulse sequences using superparamagnetic iron oxide (SHU-555-A). Abdom. Imaging 25 (2), 151–158. 10.1007/s002619910035 10675457

[B7] Bakrania A. Zheng G. Bhat M. (2021). Nanomedicine in hepatocellular carcinoma: a new frontier in targeted cancer treatment. Pharmaceutics 14 (1), 41. 10.3390/pharmaceutics14010041 35056937 PMC8779722

[B8] Beik J. Abed Z. Ghoreishi F. S. Hosseini-Nami S. Mehrzadi S. Shakeri-Zadeh A. (2016). Nanotechnology in hyperthermia cancer therapy: from fundamental principles to advanced applications. J. Control Release 235, 205–221. 10.1016/j.jconrel.2016.05.062 27264551

[B9] Bhattacharya S. Parihar V. K. Prajapati B. G. (2023). Unveiling the therapeutic potential of cabozantinib-loaded poly D,L-lactic-co-glycolic acid and polysarcosine nanoparticles in inducing apoptosis and cytotoxicity in human HepG2 hepatocellular carcinoma cell lines and *in vivo* anti-tumor activity in SCID female mice. Front. Oncol. 13, 1125857. 10.3389/fonc.2023.1125857 36874145 PMC9975495

[B10] Boateng F. Ngwa W. (2019). Delivery of nanoparticle-based radiosensitizers for radiotherapy applications. Int. J. Mol. Sci. 21 (1), 273. 10.3390/ijms21010273 31906108 PMC6981554

[B11] Bortot B. Mangogna A. Di Lorenzo G. Stabile G. Ricci G. Biffi S. (2023). Image-guided cancer surgery: a narrative review on imaging modalities and emerging nanotechnology strategies. J. Nanobiotechnology 21 (1), 155. 10.1186/s12951-023-01926-y 37202750 PMC10193783

[B12] Brown Z. J. Tsilimigras D. I. Ruff S. M. Mohseni A. Kamel I. R. Cloyd J. M. (2023). Management of hepatocellular carcinoma: a review. JAMA Surg. 158 (4), 410–420. 10.1001/jamasurg.2022.7989 36790767

[B13] Cai H. Zhu X. D. Ao J. Y. Ye B. G. Zhang Y. Y. Chai Z. T. (2017). Colony-stimulating factor-1-induced AIF1 expression in tumor-associated macrophages enhances the progression of hepatocellular carcinoma. Oncoimmunology 6 (9), e1333213. 10.1080/2162402x.2017.1333213 28932635 PMC5599077

[B14] Cai W. Chen X. (2006). Anti-angiogenic cancer therapy based on integrin alphavbeta3 antagonism. Anticancer Agents Med. Chem. 6 (5), 407–428. 10.2174/187152006778226530 17017851

[B15] Chen C. Wang Z. Ding Y. Qin Y. (2023a). Tumor microenvironment-mediated immune evasion in hepatocellular carcinoma. Front. Immunol. 14, 1133308. 10.3389/fimmu.2023.1133308 36845131 PMC9950271

[B16] Chen K. Wu Z. Zang M. Wang C. Wang Y. Wang D. (2018). Immunization with glypican-3 nanovaccine containing TLR7 agonist prevents the development of carcinogen-induced precancerous hepatic lesions to cancer in a murine model. Am. J. Transl. Res. 10 (6), 1736–1749. 30018715 PMC6038065

[B17] Chen R. C. Lii J. M. Chou C. T. Chang T. A. Chen W. T. Li C. S. (2008). T2-weighted and T1-weighted dynamic superparamagnetic iron oxide (ferucarbotran) enhanced MRI of hepatocellular carcinoma and hyperplastic nodules. J. Formos. Med. Assoc. 107 (10), 798–805. 10.1016/s0929-6646(08)60193-x 18926947

[B18] Chen S. Akbar S. M. Tanimoto K. Ninomiya T. Iuchi H. Michitaka K. (2000). Absence of CD83-positive mature and activated dendritic cells at cancer nodules from patients with hepatocellular carcinoma: relevance to hepatocarcinogenesis. Cancer Lett. 148 (1), 49–57. 10.1016/s0304-3835(99)00312-2 10680592

[B19] Chen Y. Liang Z. Lai M. (2024). Targeting the devil: strategies against cancer-associated fibroblasts in colorectal cancer. Transl. Res. 270, 81–93. 10.1016/j.trsl.2024.04.003 38614213

[B20] Chen Y. Liu S. Liao Y. Yang H. Chen Z. Hu Y. (2023b). Albumin-modified gold nanoparticles as novel radiosensitizers for enhancing lung cancer radiotherapy. Int. J. Nanomedicine 18, 1949–1964. 10.2147/ijn.S398254 37070100 PMC10105590

[B21] Cheng X. Yong Y. Dai Y. Song X. Yang G. Pan Y. (2017). Enhanced radiotherapy using bismuth sulfide nanoagents combined with photo-thermal treatment. Theranostics 7 (17), 4087–4098. 10.7150/thno.20548 29158812 PMC5694999

[B22] Cheng Y. Zhao P. Wu S. Yang T. Chen Y. Zhang X. (2018). Cisplatin and curcumin co-loaded nano-liposomes for the treatment of hepatocellular carcinoma. Int. J. Pharm. 545 (1-2), 261–273. 10.1016/j.ijpharm.2018.05.007 29730175

[B23] Chiang C. F. Hsu Y. H. Hsieh W. Y. Liao T. H. Chen C. L. Chen Y. C. (2023). IOP injection, A novel superparamagnetic iron oxide particle MRI contrast agent for the detection of hepatocellular carcinoma: a phase II clinical trial. J. Magn. Reson Imaging 58 (4), 1177–1188. 10.1002/jmri.28645 36773005

[B24] Curotto de Lafaille M. A. Lafaille J. J. (2009). Natural and adaptive foxp3+ regulatory T cells: more of the same or a division of labor? Immunity 30 (5), 626–635. 10.1016/j.immuni.2009.05.002 19464985

[B25] Da X. Cao B. Mo J. Xiang Y. Hu H. Qiu C. (2024). Inhibition of growth of hepatocellular carcinoma by co-delivery of anti-PD-1 antibody and sorafenib using biomimetic nano-platelets. BMC Cancer 24 (1), 273. 10.1186/s12885-024-12006-1 38409035 PMC10898182

[B26] Dai X. Ruan J. Guo Y. Sun Z. Liu J. Bao X. (2021). Enhanced radiotherapy efficacy and induced anti-tumor immunity in HCC by improving hypoxia microenvironment using oxygen microcapsules. Chem. Eng. J. 422, 130109. 10.1016/j.cej.2021.130109

[B27] Deng L. He K. Pan Y. Wang H. Luo Y. Xia Q. (2021). The role of tumor-associated macrophages in primary hepatocellular carcinoma and its related targeting therapy. Int. J. Med. Sci. 18 (10), 2109–2116. 10.7150/ijms.56003 33859517 PMC8040428

[B28] Deng M. Li S. H. Guo R. P. (2023). Recent advances in local thermal ablation therapy for hepatocellular carcinoma. Am. Surg. 89 (5), 1966–1973. 10.1177/00031348211054532 34743609

[B29] Donne R. Lujambio A. (2023). The liver cancer immune microenvironment: therapeutic implications for hepatocellular carcinoma. Hepatology 77 (5), 1773–1796. 10.1002/hep.32740 35989535 PMC9941399

[B30] Duan W. Liu Y. (2018). Targeted and synergistic therapy for hepatocellular carcinoma: monosaccharide modified lipid nanoparticles for the co-delivery of doxorubicin and sorafenib. Drug Des. Devel Ther. 12, 2149–2161. 10.2147/dddt.S166402 PMC6047861 30034219

[B31] Dutta R. Mahato R. I. (2017). Recent advances in hepatocellular carcinoma therapy. Pharmacol. Ther. 173, 106–117. 10.1016/j.pharmthera.2017.02.010 28174094 PMC5777523

[B32] El Dika I. Lim H. Y. Yong W. P. Lin C. C. Yoon J. H. Modiano M. (2019). An open-label, multicenter, phase I, dose escalation study with phase II expansion cohort to determine the safety, pharmacokinetics, and preliminary antitumor activity of intravenous TKM-080301 in subjects with advanced hepatocellular carcinoma. Oncologist 24 (6), 747–e218. 10.1634/theoncologist.2018-0838 30598500 PMC6656521

[B33] Elliott L. A. Doherty G. A. Sheahan K. Ryan E. J. (2017). Human tumor-infiltrating myeloid cells: phenotypic and functional diversity. Front. Immunol. 8, 86. 10.3389/fimmu.2017.00086 28220123 PMC5292650

[B34] Elnawasany S. Haggag Y. A. Shalaby S. M. Soliman N. A. El Saadany A. A. Ibrahim M. A. A. (2023). Anti-cancer effect of nano-encapsulated boswellic acids, curcumin and naringenin against HepG-2 cell line. BMC Complement. Med. Ther. 23 (1), 270. 10.1186/s12906-023-04096-4 37516826 PMC10386659

[B35] Elsayed M. M. Mostafa M. E. Alaaeldin E. Sarhan H. A. Shaykoon M. S. Allam S. (2019). Design and characterisation of novel sorafenib-loaded carbon nanotubes with distinct tumour-suppressive activity in hepatocellular carcinoma. Int. J. Nanomedicine 14, 8445–8467. 10.2147/ijn.S223920 31754301 PMC6825507

[B36] Elsayed M. M. A. Okda T. M. Atwa G. M. K. Omran G. A. Abd Elbaky A. E. Ramadan A. E. H. (2021). Design and optimization of orally administered luteolin nanoethosomes to enhance its anti-tumor activity against hepatocellular carcinoma. Pharmaceutics 13 (5), 648. 10.3390/pharmaceutics13050648 34063274 PMC8147467

[B37] Frangioni J. V. (2003). *In vivo* near-infrared fluorescence imaging. Curr. Opin. Chem. Biol. 7 (5), 626–634. 10.1016/j.cbpa.2003.08.007 14580568

[B38] Fu J. Xu D. Liu Z. Shi M. Zhao P. Fu B. (2007). Increased regulatory T cells correlate with CD8 T-cell impairment and poor survival in hepatocellular carcinoma patients. Gastroenterology 132 (7), 2328–2339. 10.1053/j.gastro.2007.03.102 17570208

[B39] Fu Q. Zhou N. Huang W. Wang D. Zhang L. Li H. (2005). Effects of nano HAP on biological and structural properties of glass bone cement. J. Biomed. Mater Res. A 74 (2), 156–163. 10.1002/jbm.a.30322 15962272

[B40] Gai X. Zhou P. Xu M. Liu Z. Zheng X. Liu Q. (2020). Hyperactivation of IL-6/STAT3 pathway leaded to the poor prognosis of post-TACE HCCs by HIF-1α/SNAI1 axis-induced epithelial to mesenchymal transition. J. Cancer 11 (3), 570–582. 10.7150/jca.35631 31942180 PMC6959052

[B41] Gao R. Gu Y. Yang Y. He Y. Huang W. Sun T. (2022). Robust radiosensitization of hemoglobin-curcumin nanoparticles suppresses hypoxic hepatocellular carcinoma. J. Nanobiotechnology 20 (1), 115. 10.1186/s12951-022-01316-w 35248069 PMC8898525

[B42] Goswami K. K. Bose A. Baral R. (2021). Macrophages in tumor: an inflammatory perspective. Clin. Immunol. 232, 108875. 10.1016/j.clim.2021.108875 34740843

[B43] Granot Z. Fridlender Z. G. (2015). Plasticity beyond cancer cells and the immunosuppressive switch. Cancer Res. 75 (21), 4441–4445. 10.1158/0008-5472.Can-15-1502 26475869

[B44] Granot Z. Jablonska J. (2015). Distinct functions of neutrophil in cancer and its regulation. Mediat. Inflamm. 2015, 701067. 10.1155/2015/701067 PMC4663337 26648665

[B45] Graur F. Puia A. Mois E. I. Moldovan S. Pusta A. Cristea C. (2022). Nanotechnology in the diagnostic and therapy of hepatocellular carcinoma. Mater. (Basel) 15 (11), 3893. 10.3390/ma15113893 PMC9182427 35683190

[B46] Guo B. Chen Q. Liu Z. Chen X. Zhu P. (2023). Adjuvant therapy following curative treatments for hepatocellular carcinoma: current dilemmas and prospects. Front. Oncol. 13, 1098958. 10.3389/fonc.2023.1098958 37139151 PMC10149944

[B47] Guo C. Hou X. Liu Y. Zhang Y. Xu H. Zhao F. (2021). Novel Chinese Angelica polysaccharide biomimetic nanomedicine to curcumin delivery for hepatocellular carcinoma treatment and immunomodulatory effect. Phytomedicine 80, 153356. 10.1016/j.phymed.2020.153356 33039729

[B48] Guo J. Zeng H. Shi X. Han T. Liu Y. Liu Y. (2022). A CFH peptide-decorated liposomal oxymatrine inactivates cancer-associated fibroblasts of hepatocellular carcinoma through epithelial-mesenchymal transition reversion. J. Nanobiotechnology 20 (1), 114. 10.1186/s12951-022-01311-1 35248071 PMC8898522

[B49] Guo M. Sun Y. Zhang X.-D. J. A. S. (2017). Enhanced radiation therapy of gold nanoparticles in liver cancer. Appl. Sci. (Basel). 7 (3), 232. 10.3390/app7030232

[B50] Han S. Bao X. Zou Y. Wang L. Li Y. Yang L. (2023). d-lactate modulates M2 tumor-associated macrophages and remodels immunosuppressive tumor microenvironment for hepatocellular carcinoma. Sci. Adv. 9 (29), eadg2697. 10.1126/sciadv.adg2697 37467325 PMC10355835

[B51] Harisinghani M. G. Saini S. Weissleder R. Halpern E. F. Schima W. Rubin D. L. (1997). Differentiation of liver hemangiomas from metastases and hepatocellular carcinoma at MR imaging enhanced with blood-pool contrast agent Code-7227. Radiology 202 (3), 687–691. 10.1148/radiology.202.3.9051017 9051017

[B52] Hato L. Vizcay A. Eguren I. Pérez-Gracia J. L. Rodríguez J. Gállego Pérez-Larraya J. (2024). Dendritic cells in cancer immunology and immunotherapy. Cancers (Basel) 16 (5), 981. 10.3390/cancers16050981 38473341 PMC10930494

[B53] He P. Xiong Y. Ye J. Chen B. Cheng H. Liu H. (2022). A clinical trial of super-stable homogeneous lipiodol-nanoICG formulation-guided precise fluorescent laparoscopic hepatocellular carcinoma resection. J. Nanobiotechnology 20 (1), 250. 10.1186/s12951-022-01467-w 35658966 PMC9164554

[B54] Hu Y. Wang S. Wu X. Zhang J. Chen R. Chen M. (2013). Chinese herbal medicine-derived compounds for cancer therapy: a focus on hepatocellular carcinoma. J. Ethnopharmacol. 149 (3), 601–612. 10.1016/j.jep.2013.07.030 23916858

[B55] Huang B. Huang M. Li Q. (2019). Cancer-associated fibroblasts promote angiogenesis of hepatocellular carcinoma by VEGF-mediated EZH2/VASH1 pathway. Technol. Cancer Res. Treat. 18, 1533033819879905. 10.1177/1533033819879905 31757187 PMC6876164

[B56] Huang Y. Ge W. Zhou J. Gao B. Qian X. Wang W. (2021). The role of tumor associated macrophages in hepatocellular carcinoma. J. Cancer 12 (5), 1284–1294. 10.7150/jca.51346 33531974 PMC7847664

[B57] Huang Y. Kou Q. Su Y. Lu L. Li X. Jiang H. (2023). Combination therapy based on dual-target biomimetic nano-delivery system for overcoming cisplatin resistance in hepatocellular carcinoma. J. Nanobiotechnology 21 (1), 89. 10.1186/s12951-023-01840-3 36918874 PMC10015699

[B58] Huang Z. Zhang Z. Jiang Y. Zhang D. Chen J. Dong L. (2012). Targeted delivery of oligonucleotides into tumor-associated macrophages for cancer immunotherapy. J. Control Release 158 (2), 286–292. 10.1016/j.jconrel.2011.11.013 22119956

[B59] Jenne C. N. Kubes P. (2013). Immune surveillance by the liver. Nat. Immunol. 14 (10), 996–1006. 10.1038/ni.2691 24048121

[B60] Jia W. Han Y. Mao X. Xu W. Zhang Y. (2022). Nanotechnology strategies for hepatocellular carcinoma diagnosis and treatment. RSC Adv. 12 (48), 31068–31082. 10.1039/d2ra05127c 36349046 PMC9621307

[B61] Jia Z. Z. Tian F. Jiang G. M. (2012). Cerebral lipiodol embolism after transarterial chemoembolization for hepatic carcinoma: a case report. World J. Gastroenterol. 18 (30), 4069–4070. 10.3748/wjg.v18.i30.4069 22912560 PMC3421436

[B62] Jin C. Bai L. Lin L. Wang S. Yin X. (2018a). Paclitaxel-loaded nanoparticles decorated with bivalent fragment HAb18 F(ab')(2) and cell penetrating peptide for improved therapeutic effect on hepatocellular carcinoma. Artif. Cells Nanomed Biotechnol. 46 (5), 1076–1084. 10.1080/21691401.2017.1360325 28776396

[B63] Jin Y. Cheng Z. Yuan Z. Du Y. Tian J. Shao B. (2024). Glucose-regulated protein 78 targeting ICG and DOX loaded hollow Fe(3)O(4) nanoparticles for hepatocellular carcinoma diagnosis and therapy. Int. J. Nanomedicine 19, 189–208. 10.2147/ijn.S428687 38223882 PMC10785830

[B64] Jin Y. Yang X. Tian J. (2018b). Targeted polypyrrole nanoparticles for the identification and treatment of hepatocellular carcinoma. Nanoscale 10 (20), 9594–9601. 10.1039/c8nr02036a 29745953

[B65] Kabakov A. E. Yakimova A. O. (2021). Hypoxia-induced cancer cell responses driving radioresistance of hypoxic tumors: approaches to targeting and radiosensitizing. Cancers (Basel) 13 (5), 1102. 10.3390/cancers13051102 33806538 PMC7961562

[B66] Kalaydina R. V. Bajwa K. Qorri B. Decarlo A. Szewczuk M. R. (2018). Recent advances in “smart” delivery systems for extended drug release in cancer therapy. Int. J. Nanomedicine 13, 4727–4745. 10.2147/ijn.S168053 30154657 PMC6108334

[B67] Kalogeridi M. A. Zygogianni A. Kyrgias G. Kouvaris J. Chatziioannou S. Kelekis N. (2015). Role of radiotherapy in the management of hepatocellular carcinoma: a systematic review. World J. Hepatol. 7 (1), 101–112. 10.4254/wjh.v7.i1.101 25625001 PMC4295187

[B68] Kang T. W. Rhim H. (2015). Recent advances in tumor ablation for hepatocellular carcinoma. Liver Cancer 4 (3), 176–187. 10.1159/000367740 26674766 PMC4608649

[B69] Keating G. M. (2017). Sorafenib: a review in hepatocellular carcinoma. Target Oncol. 12 (2), 243–253. 10.1007/s11523-017-0484-7 28299600

[B70] Khaled A. M. Othman M. S. Obeidat S. T. Aleid G. M. Aboelnaga S. M. Fehaid A. (2024). Green-synthesized silver and selenium nanoparticles using berberine: a comparative assessment of *in vitro* anticancer potential on human hepatocellular carcinoma cell line (HepG2). Cells 13 (3), 287. 10.3390/cells13030287 38334679 PMC10854975

[B71] Khan I. Saeed K. Khan I. J. A. j.o.c. (2019). Nanoparticles: properties, applications and toxicities. Arab. J. Chem. 12 (7), 908–931. 10.1016/j.arabjc.2017.05.011

[B72] Kim S. K. Kim S. H. Lee W. J. Kim H. Seo J. W. Choi D. (2002). Preoperative detection of hepatocellular carcinoma: ferumoxides-enhanced versus mangafodipir trisodium-enhanced MR imaging. AJR Am. J. Roentgenol. 179 (3), 741–750. 10.2214/ajr.179.3.1790741 12185056

[B73] Kim Y. S. Lim H. K. Rhim H. Lee M. W. (2014). Ablation of hepatocellular carcinoma. Best. Pract. Res. Clin. Gastroenterol. 28 (5), 897–908. 10.1016/j.bpg.2014.08.011 25260316

[B74] Kishore S. A. Bajwa R. Madoff D. C. (2020). Embolotherapeutic strategies for hepatocellular carcinoma: 2020 update. Cancers (Basel) 12 (4), 791. 10.3390/cancers12040791 32224882 PMC7226474

[B75] Kobayashi N. Hiraoka N. Yamagami W. Ojima H. Kanai Y. Kosuge T. (2007). FOXP3+ regulatory T cells affect the development and progression of hepatocarcinogenesis. Clin. Cancer Res. 13 (3), 902–911. 10.1158/1078-0432.Ccr-06-2363 17289884

[B76] Kobayashi T. Aikata H. Kobayashi T. Ohdan H. Arihiro K. Chayama K. (2017). Patients with early recurrence of hepatocellular carcinoma have poor prognosis. Hepatobiliary Pancreat. Dis. Int. 16 (3), 279–288. 10.1016/s1499-3872(16)60181-9 28603096

[B77] Kong F. H. Ye Q. F. Miao X. Y. Liu X. Huang S. Q. Xiong L. (2021). Current status of sorafenib nanoparticle delivery systems in the treatment of hepatocellular carcinoma. Theranostics 11 (11), 5464–5490. 10.7150/thno.54822 33859758 PMC8039945

[B78] Ladju R. B. Ulhaq Z. S. Soraya G. V. (2022). Nanotheranostics: a powerful next-generation solution to tackle hepatocellular carcinoma. World J. Gastroenterol. 28 (2), 176–187. 10.3748/wjg.v28.i2.176 35110943 PMC8776531

[B79] Lewis S. Dawson L. Barry A. Stanescu T. Mohamad I. Hosni A. (2022). Stereotactic body radiation therapy for hepatocellular carcinoma: from infancy to ongoing maturity. JHEP Rep. 4 (8), 100498. 10.1016/j.jhepr.2022.100498 35860434 PMC9289870

[B80] Li A. Tyson J. Patel S. Patel M. Katakam S. Mao X. (2021). Emerging nanotechnology for treatment of Alzheimer's and Parkinson's disease. Front. Bioeng. Biotechnol. 9, 672594. 10.3389/fbioe.2021.672594 34113606 PMC8185219

[B81] Li G. Ye L. Pan J. Long M. Zhao Z. Yang H. (2012). Antitumoural hydroxyapatite nanoparticles-mediated hepatoma-targeted trans-arterial embolization gene therapy: *in vitro* and *in vivo* studies. Liver Int. 32 (6), 998–1007. 10.1111/j.1478-3231.2012.02761.x 22340582

[B82] Li J. Liu Y. Zheng R. Qu C. Li J. (2024). Molecular mechanisms of TACE refractoriness: directions for improvement of the TACE procedure. Life Sci. 342, 122540. 10.1016/j.lfs.2024.122540 38428568

[B83] Li Y. Wang R. Xiong S. Wang X. Zhao Z. Bai S. (2019). Cancer-associated fibroblasts promote the stemness of CD24(+) liver cells via paracrine signaling. J. Mol. Med. Berl. 97 (2), 243–255. 10.1007/s00109-018-1731-9 30564864

[B84] Li Y. A. Zhao X. D. Yin H. P. Chen G. J. Yang S. Dong Y. B. (2016). A drug-loaded nanoscale metal-organic framework with a tumor targeting agent for highly effective hepatoma therapy. Chem. Commun. (Camb) 52 (98), 14113–14116. 10.1039/c6cc07321b 27858003

[B85] Liang Z. Wang Y. Wang J. Xu T. Ma S. Liu Q. (2023). Multifunctional Fe(3)O(4)-PEI@HA nanoparticles in the ferroptosis treatment of hepatocellular carcinoma through modulating reactive oxygen species. Colloids Surf. B Biointerfaces 227, 113358. 10.1016/j.colsurfb.2023.113358 37207386

[B86] Liu J. Chen J. Liu H. Zhang K. Zeng Q. Yang S. (2021). Bi/Se-Based nanotherapeutics sensitize CT image-guided stereotactic body radiotherapy through reprogramming the microenvironment of hepatocellular carcinoma. ACS Appl. Mater Interfaces 13 (36), 42473–42485. 10.1021/acsami.1c11763 34474563

[B87] Liu J. Mu W. Gao T. Fang Y. Zhang N. Liu Y. (2023). CD13-Mediated pegylated carboxymethyl chitosan-capped mesoporous silica nanoparticles for enhancing the therapeutic efficacy of hepatocellular carcinoma. Pharmaceutics 15 (2), 426. 10.3390/pharmaceutics15020426 36839748 PMC9962034

[B88] Liu Q. Zhu H. Liu Y. Musetti S. Huang L. (2018). BRAF peptide vaccine facilitates therapy of murine BRAF-mutant melanoma. Cancer Immunol. Immunother. 67 (2), 299–310. 10.1007/s00262-017-2079-7 29094184 PMC5801101

[B89] Llovet J. M. Fuster J. Bruix J. Barcelona-Clínic Liver Cancer Group (2004). The Barcelona approach: diagnosis, staging, and treatment of hepatocellular carcinoma. Liver Transpl. 10 (2 Suppl. 1), S115–S120. 10.1002/lt.20034 14762851

[B90] Lu C. Rong D. Zhang B. Zheng W. Wang X. Chen Z. (2019). Current perspectives on the immunosuppressive tumor microenvironment in hepatocellular carcinoma: challenges and opportunities. Mol. Cancer 18 (1), 130. 10.1186/s12943-019-1047-6 31464625 PMC6714090

[B91] Lu S. Zhang C. Wang J. Zhao L. Li G. (2024). Research progress in nano-drug delivery systems based on the characteristics of the liver cancer microenvironment. Biomed. Pharmacother. 170, 116059. 10.1016/j.biopha.2023.116059 38154273

[B92] Lyu N. Lin Y. Kong Y. Zhang Z. Liu L. Zheng L. (2018). FOXAI: a phase II trial evaluating the efficacy and safety of hepatic arterial infusion of oxaliplatin plus fluorouracil/leucovorin for advanced hepatocellular carcinoma. Gut 67 (2), 395–396. 10.1136/gutjnl-2017-314138 28592441

[B93] Malik S. Waheed Y. (2023). Emerging applications of nanotechnology in dentistry. Dent. J. (Basel) 11 (11), 266. 10.3390/dj11110266 37999030 PMC10670129

[B94] Mergo P. J. Engelken J. D. Helmberger T. Ros P. R. (1998). MRI in focal liver disease: a comparison of small and ultra-small superparamagnetic iron oxide as hepatic contrast agents. J. Magn. Reson Imaging 8 (5), 1073–1078. 10.1002/jmri.1880080511 9786144

[B95] Miao T. G. Nan Y. M. (2022). Hepatocellular carcinoma immune microenvironment. Zhonghua Gan Zang Bing Za Zhi 30 (9), 923–930. 10.3760/cma.j.cn501113-20220703-00365 36299184 PMC12770770

[B96] Mondal J. Khuda-Bukhsh A. R. (2020). Cisplatin and farnesol co-encapsulated PLGA nano-particles demonstrate enhanced anti-cancer potential against hepatocellular carcinoma cells *in vitro* . Mol. Biol. Rep. 47 (5), 3615–3628. 10.1007/s11033-020-05455-x 32314187

[B97] Nagaraj S. Gupta K. Pisarev V. Kinarsky L. Sherman S. Kang L. (2007). Altered recognition of antigen is a mechanism of CD8+ T cell tolerance in cancer. Nat. Med. 13 (7), 828–835. 10.1038/nm1609 17603493 PMC2135607

[B98] Nel A. Xia T. Mädler L. Li N. (2006). Toxic potential of materials at the nanolevel. Science 311 (5761), 622–627. 10.1126/science.1114397 16456071

[B99] Nevola R. Tortorella G. Rosato V. Rinaldi L. Imbriani S. Perillo P. (2023). Gender differences in the pathogenesis and risk factors of hepatocellular carcinoma. Biol. (Basel) 12 (7), 984. 10.3390/biology12070984 PMC10376683 37508414

[B100] Niveria K. Yadav M. Dangi K. Verma A. K. J. O. (2022) Overcoming challenges to enable targeting of metastatic breast cancer tumour microenvironment with nano-therapeutics: current status and future perspectives, OpenNano 8.100083. 10.1016/j.onano.2022.100083

[B101] Oroojalian F. Beygi M. Baradaran B. Mokhtarzadeh A. Shahbazi M. A. (2021). Immune cell membrane-coated biomimetic nanoparticles for targeted cancer therapy. Small 17 (12), e2006484. 10.1002/smll.202006484 33577127

[B102] Osuga K. Maeda N. Higashihara H. Hori S. Nakazawa T. Tanaka K. (2012). Current status of embolic agents for liver tumor embolization. Int. J. Clin. Oncol. 17 (4), 306–315. 10.1007/s10147-012-0445-1 22806426

[B103] Ou W. Thapa R. K. Jiang L. Soe Z. C. Gautam M. Chang J. H. (2018). Regulatory T cell-targeted hybrid nanoparticles combined with immuno-checkpoint blockage for cancer immunotherapy. J. Control Release 281, 84–96. 10.1016/j.jconrel.2018.05.018 29777794

[B104] Oura K. Morishita A. Tani J. Masaki T. (2021). Tumor immune microenvironment and immunosuppressive therapy in hepatocellular carcinoma: a review. Int. J. Mol. Sci. 22 (11), 5801. 10.3390/ijms22115801 34071550 PMC8198390

[B105] Pan X. Ni S. Hu K. (2024). Nanomedicines for reversing immunosuppressive microenvironment of hepatocellular carcinoma. Biomaterials 306, 122481. 10.1016/j.biomaterials.2024.122481 38286109

[B106] Pandey P. Rahman M. Bhatt P. C. Beg S. Paul B. Hafeez A. (2018). Implication of nano-antioxidant therapy for treatment of hepatocellular carcinoma using PLGA nanoparticles of rutin. Nanomedicine (Lond) 13 (8), 849–870. 10.2217/nnm-2017-0306 29565220

[B107] Peng Y. He P. Gao X. Liu G. Cheng H. (2022). A superstable homogeneous lipiodol-nanoformulation to overcome the dilemma of interventional embolization chemotherapy. Front. Bioeng. Biotechnol. 10, 952194. 10.3389/fbioe.2022.952194 35800328 PMC9253561

[B108] Pérez-Romasanta L. A. González-Del Portillo E. Rodríguez-Gutiérrez A. Matías-Pérez Á. (2021). Stereotactic radiotherapy for hepatocellular carcinoma, radiosensitization strategies and radiation-immunotherapy combination. Cancers (Basel) 13 (2), 192. 10.3390/cancers13020192 33430362 PMC7825787

[B109] Persson E. C. Schwartz L. M. Park Y. Trabert B. Hollenbeck A. R. Graubard B. I. (2013). Alcohol consumption, folate intake, hepatocellular carcinoma, and liver disease mortality. Cancer Epidemiol. Biomarkers Prev. 22 (3), 415–421. 10.1158/1055-9965.Epi-12-1169 23307533 PMC3596467

[B110] Petrick J. L. Campbell P. T. Koshiol J. Thistle J. E. Andreotti G. Beane-Freeman L. E. (2018). Tobacco, alcohol use and risk of hepatocellular carcinoma and intrahepatic cholangiocarcinoma: the Liver Cancer Pooling Project. Br. J. Cancer 118 (7), 1005–1012. 10.1038/s41416-018-0007-z 29520041 PMC5931109

[B111] Qian J. Oppermann E. Tran A. Imlau U. Qian K. Vogl T. J. (2016). Transarterial administration of integrin inhibitor loaded nanoparticles combined with transarterial chemoembolization for treating hepatocellular carcinoma in a rat model. World J. Gastroenterol. 22 (21), 5042–5049. 10.3748/wjg.v22.i21.5042 27275096 PMC4886379

[B112] Que H. Fu Q. Lan T. Tian X. Wei X. (2022). Tumor-associated neutrophils and neutrophil-targeted cancer therapies. Biochim. Biophys. Acta Rev. Cancer 1877 (5), 188762. 10.1016/j.bbcan.2022.188762 35853517

[B113] Rahman M. Al-Ghamdi S. A. Alharbi K. S. Beg S. Sharma K. Anwar F. (2019). Ganoderic acid loaded nano-lipidic carriers improvise treatment of hepatocellular carcinoma. Drug Deliv. 26 (1), 782–793. 10.1080/10717544.2019.1606865 31357897 PMC6711158

[B114] Rancoule C. Magné N. Vallard A. Guy J. B. Rodriguez-Lafrasse C. Deutsch E. (2016). Nanoparticles in radiation oncology: from bench-side to bedside. Cancer Lett. 375 (2), 256–262. 10.1016/j.canlet.2016.03.011 26987625

[B115] Sagiv J. Y. Michaeli J. Assi S. Mishalian I. Kisos H. Levy L. (2015). Phenotypic diversity and plasticity in circulating neutrophil subpopulations in cancer. Cell Rep. 10 (4), 562–573. 10.1016/j.celrep.2014.12.039 25620698

[B116] Saleh T. Shojaosadati S. A. (2016). Multifunctional nanoparticles for cancer immunotherapy. Hum. Vaccin Immunother. 12 (7), 1863–1875. 10.1080/21645515.2016.1147635 26901287 PMC4964832

[B117] Saravanakumar K. Anbazhagan S. Pujani Usliyanage J. Vishven Naveen K. Wijesinghe U. Xiaowen H. (2022). A comprehensive review on immuno-nanomedicine for breast cancer therapy: technical challenges and troubleshooting measures. Int. Immunopharmacol. 103, 108433. 10.1016/j.intimp.2021.108433 34922248

[B118] Schwendener R. A. (2014). Liposomes as vaccine delivery systems: a review of the recent advances. Ther. Adv. Vaccines 2 (6), 159–182. 10.1177/2051013614541440 25364509 PMC4212474

[B119] Seyfoori A. Shokrollahi Barough M. Mokarram P. Ahmadi M. Mehrbod P. Sheidary A. (2021). Emerging advances of nanotechnology in drug and vaccine delivery against viral associated respiratory infectious diseases (VARID). Int. J. Mol. Sci. 22 (13), 6937. 10.3390/ijms22136937 34203268 PMC8269337

[B120] Sharma B. Crist R. M. Adiseshaiah P. P. (2017). Nanotechnology as a delivery tool for precision cancer therapies. Aaps J. 19 (6), 1632–1642. 10.1208/s12248-017-0152-y 29019032

[B121] Shaul M. E. Fridlender Z. G. (2018). Cancer-related circulating and tumor-associated neutrophils - subtypes, sources and function. Febs J. 285 (23), 4316–4342. 10.1111/febs.14524 29851227

[B122] Shi C. Chen Y. Chen Y. Yang Y. Bing W. Qi J. (2019). CD4^+^ CD25^+^ regulatory T cells promote hepatocellular carcinoma invasion via TGF-β1-induced epithelial-mesenchymal transition. Onco Targets Ther. 12, 279–289. 10.2147/ott.S172417 30643426 PMC6314313

[B123] Shiri A. M. Zhang T. Bedke T. Zazara D. E. Zhao L. Lücke J. (2024). IL-10 dampens antitumor immunity and promotes liver metastasis via PD-L1 induction. J. Hepatol. 80 (4), 634–644. 10.1016/j.jhep.2023.12.015 38160941 PMC10964083

[B124] Simon T. Salhia B. (2022). Cancer-associated fibroblast subpopulations with diverse and dynamic roles in the tumor microenvironment. Mol. Cancer Res. 20 (2), 183–192. 10.1158/1541-7786.Mcr-21-0282 34670861 PMC9306405

[B125] Sionov R. V. Fridlender Z. G. Granot Z. (2015). The multifaceted roles neutrophils play in the tumor microenvironment. Cancer Microenviron. 8 (3), 125–158. 10.1007/s12307-014-0147-5 24895166 PMC4714999

[B126] Somasundaram V. H. Pillai R. Malarvizhi G. Ashokan A. Gowd S. Peethambaran R. (2016). Biodegradable radiofrequency responsive nanoparticles for augmented thermal ablation combined with triggered drug release in liver tumors. ACS Biomater. Sci. Eng. 2 (5), 768–779. 10.1021/acsbiomaterials.5b00511 33440574

[B127] Song N. Sun S. Chen K. Wang Y. Wang H. Meng J. (2023a). Emerging nanotechnology for Alzheimer's disease: from detection to treatment. J. Control Release 360, 392–417. 10.1016/j.jconrel.2023.07.004 37414222

[B128] Song X. Sun Z. Li L. Zhou L. Yuan S. (2023b). Application of nanomedicine in radiotherapy sensitization. Front. Oncol. 13, 1088878. 10.3389/fonc.2023.1088878 36874097 PMC9977159

[B129] Su D. (2021). The transcatheter arterial chemoembolization combined with targeted nanoparticle delivering sorafenib system for the treatment of microvascular invasion of hepatocellular carcinoma. Bioengineered 12 (2), 11124–11135. 10.1080/21655979.2021.2001239 34923912 PMC8810100

[B130] Sukhanova A. Bozrova S. Sokolov P. Berestovoy M. Karaulov A. Nabiev I. (2018). Dependence of nanoparticle toxicity on their physical and chemical properties. Nanoscale Res. Lett. 13 (1), 44. 10.1186/s11671-018-2457-x 29417375 PMC5803171

[B131] Tan L. Chen S. Wei G. Li Y. Liao J. Jin H. (2019). Sublethal heat treatment of hepatocellular carcinoma promotes intrahepatic metastasis and stemness in a VEGFR1-dependent manner. Cancer Lett. 460, 29–40. 10.1016/j.canlet.2019.05.041 31173855

[B132] Tang M. Huang Y. Liang X. Tao Y. He N. Li Z. (2022). Sorafenib-loaded PLGA-TPGS nanosystems enhance hepatocellular carcinoma therapy through reversing P-Glycoprotein-Mediated multidrug resistance. AAPS PharmSciTech 23 (5), 130. 10.1208/s12249-022-02214-y 35487999

[B133] Tang W. Chen Z. Zhang W. Cheng Y. Zhang B. Wu F. (2020a). The mechanisms of sorafenib resistance in hepatocellular carcinoma: theoretical basis and therapeutic aspects. Signal Transduct. Target Ther. 5 (1), 87. 10.1038/s41392-020-0187-x 32532960 PMC7292831

[B134] Tang Y. Shu Z. Zhu M. Li S. Ling Y. Fu Y. (2023). Size-tunable nanoregulator-based radiofrequency ablation suppresses MDSCs and their compensatory immune evasion in hepatocellular carcinoma. Adv. Healthc. Mater 12 (30), e2302013. 10.1002/adhm.202302013 37665720

[B135] Tang Z. Luo C. Jun Y. Yao M. Zhang M. He K. (2020b). Nanovector assembled from natural egg yolk lipids for tumor-targeted delivery of therapeutics. ACS Appl. Mater Interfaces 12 (7), 7984–7994. 10.1021/acsami.9b22293 31971362

[B136] Taniai M. (2020). Alcohol and hepatocarcinogenesis. Clin. Mol. Hepatol. 26 (4), 736–741. 10.3350/cmh.2020.0203 33053943 PMC7641552

[B137] Thai S. F. Jones C. P. Robinette B. L. Ren H. Vallanat B. Fisher A. A. (2021). Effects of silver nanoparticles and silver nitrate on mRNA and microRNA expression in human hepatocellular carcinoma cells (HepG2). J. Nanosci. Nanotechnol. 21 (11), 5414–5428. 10.1166/jnn.2021.19481 33980351 PMC10563035

[B138] Tomiyama T. Itoh S. Iseda N. Toshida K. Morinaga A. Yugawa K. (2022). Myeloid-derived suppressor cell infiltration is associated with a poor prognosis in patients with hepatocellular carcinoma. Oncol. Lett. 23 (3), 93. 10.3892/ol.2022.13213 35154424 PMC8822414

[B139] Topalian S. L. Weiner G. J. Pardoll D. M. (2011). Cancer immunotherapy comes of age. J. Clin. Oncol. 29 (36), 4828–4836. 10.1200/jco.2011.38.0899 22042955 PMC3255990

[B140] Umansky V. Blattner C. Gebhardt C. Utikal J. (2016). The role of myeloid-derived suppressor cells (MDSC) in cancer progression. Vaccines (Basel) 4 (4), 36. 10.3390/vaccines4040036 27827871 PMC5192356

[B141] Unnisa A. Greig N. H. Kamal M. A. (2023). Nanotechnology: a promising targeted drug delivery system for brain tumours and Alzheimer's disease. Curr. Med. Chem. 30 (3), 255–270. 10.2174/0929867329666220328125206 35345990 PMC11335033

[B142] Verbeek F. P. van der Vorst J. R. Schaafsma B. E. Hutteman M. Bonsing B. A. van Leeuwen F. W. (2012). Image-guided hepatopancreatobiliary surgery using near-infrared fluorescent light. J. Hepatobiliary Pancreat. Sci. 19 (6), 626–637. 10.1007/s00534-012-0534-6 22790312 PMC3501168

[B143] Verry C. Sancey L. Dufort S. Le Duc G. Mendoza C. Lux F. (2019). Treatment of multiple brain metastases using gadolinium nanoparticles and radiotherapy: NANO-RAD, a phase I study protocol. BMJ Open 9 (2), e023591. 10.1136/bmjopen-2018-023591 PMC6377538 30755445

[B144] Wang B. Sun L. Wen M. Tan Y. Almalki W. H. Katouah H. (2021a). Nano lipidic carriers for codelivery of sorafenib and ganoderic acid for enhanced synergistic antitumor efficacy against hepatocellular carcinoma. Saudi Pharm. J. 29 (8), 843–856. 10.1016/j.jsps.2021.06.006 34408545 PMC8363106

[B145] Wang S. Wu Q. Chen T. Su R. Pan C. Qian J. (2022a). Blocking CD47 promotes antitumour immunity through CD103(+) dendritic cell-NK cell axis in murine hepatocellular carcinoma model. J. Hepatol. 77 (2), 467–478. 10.1016/j.jhep.2022.03.011 35367532

[B146] Wang T. Zhang J. Hou T. Yin X. Zhang N. (2019). Selective targeting of tumor cells and tumor associated macrophages separately by twin-like core-shell nanoparticles for enhanced tumor-localized chemoimmunotherapy. Nanoscale 11 (29), 13934–13946. 10.1039/c9nr03374b 31305839

[B147] Wang Y. Zhang T. Sun M. Ji X. Xie M. Huang W. (2021b). Therapeutic values of myeloid-derived suppressor cells in hepatocellular carcinoma: facts and hopes. Cancers (Basel) 13 (20), 5127. 10.3390/cancers13205127 34680276 PMC8534227

[B148] Wang Y. Zhao Q. Zhao B. Zheng Y. Zhuang Q. Liao N. (2022b). Remodeling tumor-associated neutrophils to enhance dendritic cell-based HCC neoantigen nano-vaccine efficiency. Adv. Sci. (Weinh) 9 (11), e2105631. 10.1002/advs.202105631 35142445 PMC9009112

[B149] Wáng Y. X. De Baere T. Idée J. M. Ballet S. (2015). Transcatheter embolization therapy in liver cancer: an update of clinical evidences. Chin. J. Cancer Res. 27 (2), 96–121. 10.3978/j.issn.1000-9604.2015.03.03 25937772 PMC4409973

[B150] Wculek S. K. Cueto F. J. Mujal A. M. Melero I. Krummel M. F. Sancho D. (2020). Dendritic cells in cancer immunology and immunotherapy. Nat. Rev. Immunol. 20 (1), 7–24. 10.1038/s41577-019-0210-z 31467405

[B151] Wei Y. Wei Y. Sheng L. Ma J. Su Z. Wen J. (2023). Construction of curcumin and paclitaxel Co-loaded lipid nano platform and evaluation of its anti-hepatoma activity *in vitro* and pharmacokinetics *in vivo* . Int. J. Nanomedicine 18, 2087–2107. 10.2147/ijn.S399289 37122500 PMC10135418

[B152] Weissleder R. Kelly K. Sun E. Y. Shtatland T. Josephson L. (2005). Cell-specific targeting of nanoparticles by multivalent attachment of small molecules. Nat. Biotechnol. 23 (11), 1418–1423. 10.1038/nbt1159 16244656

[B153] Wojtynek N. E. Mohs A. M. (2020). Image-guided tumor surgery: the emerging role of nanotechnology. Wiley Interdiscip. Rev. Nanomed Nanobiotechnol 12 (4), e1624. 10.1002/wnan.1624 32162485 PMC9469762

[B154] Wu H. Wang M. D. Liang L. Xing H. Zhang C. W. Shen F. (2021). Nanotechnology for hepatocellular carcinoma: from surveillance, diagnosis to management. Small 17 (6), e2005236. 10.1002/smll.202005236 33448111

[B155] Wu J. Qi C. Wang H. Wang Q. Sun J. Dong J. (2022). Curcumin and berberine co-loaded liposomes for anti-hepatocellular carcinoma therapy by blocking the cross-talk between hepatic stellate cells and tumor cells. Front. Pharmacol. 13, 961788. 10.3389/fphar.2022.961788 36188590 PMC9515508

[B156] Wu L. P. Wang D. Li Z. (2020a). Grand challenges in nanomedicine. Mater Sci. Eng. C Mater Biol. Appl. 106, 110302. 10.1016/j.msec.2019.110302 31753337

[B157] Wu S. Fan K. Yang Q. Chen Z. Hou Y. Zou Y. (2023). Smart nanoparticles and microbeads for interventional embolization therapy of liver cancer: state of the art. J. Nanobiotechnology 21 (1), 42. 10.1186/s12951-023-01804-7 36747202 PMC9901004

[B158] Wu S. Zhang D. Yu J. Dou J. Li X. Mu M. (2020b). Chemotherapeutic nanoparticle-based liposomes enhance the efficiency of Mild microwave ablation in hepatocellular carcinoma therapy. Front. Pharmacol. 11, 85. 10.3389/fphar.2020.00085 32174827 PMC7054279

[B159] Xiao Z. Li T. Zheng X. Lin L. Wang X. Li B. (2023). Nanodrug enhances post-ablation immunotherapy of hepatocellular carcinoma via promoting dendritic cell maturation and antigen presentation. Bioact. Mater 21, 57–68. 10.1016/j.bioactmat.2022.07.027 36017073 PMC9399385

[B160] Xu W. Yang M. Zhang W. Jia W. Zhang H. Zhang Y. (2024). Tumor microenvironment responsive nano-platform for overcoming sorafenib resistance of hepatocellular carcinoma. Mater Today Bio 24, 100902. 10.1016/j.mtbio.2023.100902 PMC10767498 38188646

[B161] Xu Y. Li X. Gong W. Huang H. B. Zhu B. W. Hu J. N. (2020). Construction of ginsenoside nanoparticles with pH/reduction dual response for enhancement of their cytotoxicity toward HepG2 cells. J. Agric. Food Chem. 68 (32), 8545–8556. 10.1021/acs.jafc.0c03698 32686932

[B162] Yamamoto H. Yamashita Y. Yoshimatsu S. Baba Y. Hatanaka Y. Murakami R. (1995). Hepatocellular carcinoma in cirrhotic livers: detection with unenhanced and iron oxide-enhanced MR imaging. Radiology 195 (1), 106–112. 10.1148/radiology.195.1.7892448 7892448

[B163] Yang F. Dai L. Shi K. Liu Q. Pan M. Mo D. (2024). A facile boronophenylalanine modified polydopamine dual drug-loaded nanoparticles for enhanced anti-tumor immune response in hepatocellular carcinoma comprehensive treatment. Biomaterials 305, 122435. 10.1016/j.biomaterials.2023.122435 38150771

[B164] Yang J. D. Mohamed E. A. Aziz A. O. Shousha H. I. Hashem M. B. Nabeel M. M. (2017). Characteristics, management, and outcomes of patients with hepatocellular carcinoma in Africa: a multicountry observational study from the Africa Liver Cancer Consortium. Lancet Gastroenterol. Hepatol. 2 (2), 103–111. 10.1016/s2468-1253(16)30161-3 28403980

[B165] Yang X. Lin Y. Shi Y. Li B. Liu W. Yin W. (2016). FAP promotes immunosuppression by cancer-associated fibroblasts in the tumor microenvironment via STAT3-CCL2 signaling. Cancer Res. 76 (14), 4124–4135. 10.1158/0008-5472.Can-15-2973 27216177

[B166] Yao R. R. Li J. H. Zhang R. Chen R. X. Wang Y. H. (2018). M2-polarized tumor-associated macrophages facilitated migration and epithelial-mesenchymal transition of HCC cells via the TLR4/STAT3 signaling pathway. World J. Surg. Oncol. 16 (1), 9. 10.1186/s12957-018-1312-y 29338742 PMC5771014

[B167] Yin Y. Feng W. Chen J. Chen X. Wang G. Wang S. (2024). Immunosuppressive tumor microenvironment in the progression, metastasis, and therapy of hepatocellular carcinoma: from bench to bedside. Exp. Hematol. Oncol. 13 (1), 72. 10.1186/s40164-024-00539-x 39085965 PMC11292955

[B168] Yin Z. Huang J. Ma T. Li D. Wu Z. Hou B. (2017). Macrophages activating chemokine (C-X-C motif) ligand 8/miR-17 cluster modulate hepatocellular carcinoma cell growth and metastasis. Am. J. Transl. Res. 9 (5), 2403–2411. 28559990 PMC5446522

[B169] Yoshikawa K. Sasaki Y. Ogawa N. Sakuma S. (1994). Clinical application of AMI-25 (superparamagnetic iron oxide) for the MR imaging of hepatic tumors: a multicenter clinical phase III study. Nihon Igaku Hoshasen Gakkai Zasshi 54 (2), 137–153. 8121779

[B170] Yousef S. Alsaab H. O. Sau S. Iyer A. K. (2018). Development of asialoglycoprotein receptor directed nanoparticles for selective delivery of curcumin derivative to hepatocellular carcinoma. Heliyon 4 (12), e01071. 10.1016/j.heliyon.2018.e01071 30603704 PMC6305692

[B171] Yu Z. Guo J. Hu M. Gao Y. Huang L. (2020). Icaritin exacerbates mitophagy and synergizes with doxorubicin to induce immunogenic cell death in hepatocellular carcinoma. ACS Nano 14 (4), 4816–4828. 10.1021/acsnano.0c00708 32188241

[B172] Yu Z. Guo J. Liu Y. Wang M. Liu Z. Gao Y. (2022). Nano delivery of simvastatin targets liver sinusoidal endothelial cells to remodel tumor microenvironment for hepatocellular carcinoma. J. Nanobiotechnology 20 (1), 9. 10.1186/s12951-021-01205-8 34983554 PMC8725360

[B173] Yuan G. Liu Z. Wang W. Liu M. Xu Y. Hu W. (2023a). Multifunctional nanoplatforms application in the transcatheter chemoembolization against hepatocellular carcinoma. J. Nanobiotechnology 21 (1), 68. 10.1186/s12951-023-01820-7 36849981 PMC9969656

[B174] Yuan G. Xu Y. Bai X. Wang W. Wu X. Chen J. (2023b). Autophagy-targeted calcium phosphate nanoparticles enable transarterial chemoembolization for enhanced cancer therapy. ACS Appl. Mater Interfaces 15 (9), 11431–11443. 10.1021/acsami.2c18267 36848495

[B175] Zeng F. Zhang Y. Han X. Zeng M. Gao Y. Weng J. (2021). Employing hypoxia characterization to predict tumour immune microenvironment, treatment sensitivity and prognosis in hepatocellular carcinoma. Comput. Struct. Biotechnol. J. 19, 2775–2789. 10.1016/j.csbj.2021.03.033 34093992 PMC8134035

[B176] Zeng J. Li L. Zhang H. Li J. Liu L. Zhou G. (2018). Radiopaque and uniform alginate microspheres loaded with tantalum nanoparticles for real-time imaging during transcatheter arterial embolization. Theranostics 8 (17), 4591–4600. 10.7150/thno.27379 30279724 PMC6160769

[B177] Zhang B. Jiang J. Wu P. Zou J. Le J. Lin J. (2021a). A smart dual-drug nanosystem based on co-assembly of plant and food-derived natural products for synergistic HCC immunotherapy. Acta Pharm. Sin. B 11 (1), 246–257. 10.1016/j.apsb.2020.07.026 33532190 PMC7838026

[B178] Zhang D. Lin Z. Zheng Y. Song J. Li J. Zeng Y. (2020a). Ultrasound-driven biomimetic nanosystem suppresses tumor growth and metastasis through sonodynamic therapy, CO therapy, and indoleamine 2,3-Dioxygenase inhibition. ACS Nano 14 (7), 8985–8999. 10.1021/acsnano.0c03833 32662971

[B179] Zhang J. Gu C. Song Q. Zhu M. Xu Y. Xiao M. (2020b). Identifying cancer-associated fibroblasts as emerging targets for hepatocellular carcinoma. Cell Biosci. 10 (1), 127. 10.1186/s13578-020-00488-y 33292459 PMC7603733

[B180] Zhang L. Huang J. Chen X. Pan C. He Y. Su R. (2021b). Self-assembly nanovaccine containing TLR7/8 agonist and STAT3 inhibitor enhances tumor immunotherapy by augmenting tumor-specific immune response. J. Immunother. Cancer 9 (8), e003132. 10.1136/jitc-2021-003132 34452929 PMC8404452

[B181] Zhang P. Zhang L. Qin Z. Hua S. Guo Z. Chu C. (2018). Genetically engineered liposome-like nanovesicles as active targeted transport platform. Adv. Mater 30 (7). 10.1002/adma.201705350 29280210

[B182] Zhang R. R. Schroeder A. B. Grudzinski J. J. Rosenthal E. L. Warram J. M. Pinchuk A. N. (2017). Beyond the margins: real-time detection of cancer using targeted fluorophores. Nat. Rev. Clin. Oncol. 14 (6), 347–364. 10.1038/nrclinonc.2016.212 28094261 PMC5683405

[B183] Zhang Y. Cheng H. Chen H. Xu P. Ren E. Jiang Y. (2022). A pure nanoICG-based homogeneous lipiodol formulation: toward precise surgical navigation of primary liver cancer after long-term transcatheter arterial embolization. Eur. J. Nucl. Med. Mol. Imaging 49 (8), 2605–2617. 10.1007/s00259-021-05654-z 34939176

[B184] Zhang Y. Xie F. Yin Y. Zhang Q. Jin H. Wu Y. (2021c). Immunotherapy of tumor RNA-loaded lipid nanoparticles against hepatocellular carcinoma. Int. J. Nanomedicine 16, 1553–1564. 10.2147/ijn.S291421 33658783 PMC7920588

[B185] Zhao H. Wang H. Q. Fan Q. Q. Chen X. X. Lou J. Y. (2008). Rare pulmonary and cerebral complications after transarterial chemoembolisation for hepatocellular carcinoma: a case report. World J. Gastroenterol. 14 (41), 6425–6427. 10.3748/wjg.14.6425 19009665 PMC2766131

[B186] Zhao Y. Yan B. Wang Z. Li M. Zhao W. (2020). Natural polysaccharides with immunomodulatory activities. Mini Rev. Med. Chem. 20 (2), 96–106. 10.2174/1389557519666190913151632 31518223

[B187] Zheng Q. Yang H. Wei J. Tong J. L. Shu Y. Q. (2013). The role and mechanisms of nanoparticles to enhance radiosensitivity in hepatocellular cell. Biomed. Pharmacother. 67 (7), 569–575. 10.1016/j.biopha.2013.04.003 23786887

[B188] Zhou J. Sun H. Wang Z. Cong W. Wang J. Zeng M. (2020). Guidelines for the diagnosis and treatment of hepatocellular carcinoma (2019 edition). Liver Cancer 9 (6), 682–720. 10.1159/000509424 33442540 PMC7768108

[B189] Zhou L. Wang J. Lyu S. C. Pan L. C. Shi X. J. Du G. S. (2021). PD-L1(+)NEUT, Foxp3(+)Treg, and NLR as new prognostic marker with low survival benefits value in hepatocellular carcinoma. Technol. Cancer Res. Treat. 20, 15330338211045820. 10.1177/15330338211045820 34605709 PMC8493317

[B190] Zhou Q. Sun X. Zeng L. Liu J. Zhang Z. (2009). A randomized multicenter phase II clinical trial of mitoxantrone-loaded nanoparticles in the treatment of 108 patients with unresected hepatocellular carcinoma. Nanomedicine 5 (4), 419–423. 10.1016/j.nano.2009.01.009 19523421

[B191] Zhou S. L. Hu Z. Q. Zhou Z. J. Dai Z. Wang Z. Cao Y. (2016). miR-28-5p-IL-34-macrophage feedback loop modulates hepatocellular carcinoma metastasis. Hepatology 63 (5), 1560–1575. 10.1002/hep.28445 26754294

[B192] Zhou S. L. Yin D. Hu Z. Q. Luo C. B. Zhou Z. J. Xin H. Y. (2019). A positive feedback loop between cancer stem-like cells and tumor-associated neutrophils controls hepatocellular carcinoma progression. Hepatology 70 (4), 1214–1230. 10.1002/hep.30630 30933361

[B193] Zhu J. Chu C. Li D. Zhang Y. Cheng Y. Lin H. (2022). Superior fluorescent nanoemulsion illuminates hepatocellular carcinoma for surgical navigation. Front. Bioeng. Biotechnol. 10, 890668. 10.3389/fbioe.2022.890668 35547157 PMC9081524

[B194] Zhu J. Q. Wu H. Li X. Li M. Y. Li Z. L. Xu X. F. (2024). Hydrogel crosslinked with nanoparticles for prevention of surgical hemorrhage and recurrence of hepatocellular carcinoma. Adv. Sci. (Weinh) 11 (9), e2305508. 10.1002/advs.202305508 38145957 PMC10916646

[B195] Zhu Y. Yu X. Thamphiwatana S. D. Zheng Y. Pang Z. (2020). Nanomedicines modulating tumor immunosuppressive cells to enhance cancer immunotherapy. Acta Pharm. Sin. B 10 (11), 2054–2074. 10.1016/j.apsb.2020.08.010 33304779 PMC7714985

